# Sixteen prescribed Chinese herbal medicines provide time-dependent cardiorenal and survival benefits in patients with overall and advanced diabetic kidney disease: a real-world study in Taiwan

**DOI:** 10.3389/fphar.2024.1297854

**Published:** 2024-08-22

**Authors:** Hsiao-Tien Chen, Chien-Hsueh Tung, Ben-Hui Yu, Yi-Chun Chen

**Affiliations:** ^1^ Department of Chinese Medicine, Chi Mei Medical Center, Tainan, Taiwan; ^2^ Division of Allergy, Immunology and Rheumatology, Department of Internal Medicine, Dalin Tzu Chi Hospital, Buddhist Tzu Chi Medical Foundation, Chiayi, Taiwan; ^3^ School of Medicine, Tzu Chi University, Hualien, Taiwan; ^4^ Department of Radiation Oncology, Dalin Tzu Chi Hospital, Buddhist Tzu Chi Medical Foundation, Chiayi, Taiwan; ^5^ Division of Nephrology, Department of Internal Medicine, Dalin Tzu Chi Hospital, Buddhist Tzu Chi Medical Foundation, Chiayi, Taiwan

**Keywords:** renoprotective Chinese herbal medicines, scientific Chinese medicine, diabetic kidney disease, oxidative stress, time-dependent, ESRD, cardiovascular mortality, all-cause mortality

## Abstract

**Background:**

A causal connection between oxidative stress and inflammation in diabetes, along with its associated renal and cardiovascular complications, has been established. Sixteen prescribed potentially renoprotective Chinese herbal medicines for diabetic kidney disease (PRCHMDKD), which are scientific Chinese medicine (botanical drug) and categorized into five classes (clearing heat, nourishing yin, dampness dispelling, tonifying qi, and harmonizing formulas), exhibit shared antioxidative properties and target multiple oxidative stress pathways. However, the time-response, cumulative effects, and safety (hyperkalemia risk) of these sixteen PRCHMDKD on cardiorenal and survival outcomes in patients with overall and advanced DKD remain unresolved.

**Methods:**

This retrospective cohort study analyzed national health insurance claims data in 2000–2017. Four statistical methods, including Cox proportional hazards models, complementary restricted mean survival time (RMST), propensity score matching, and competing risk analysis for end-stage renal disease (ESRD), were employed to investigate this relationship. The study included 43,480 PRCHMDKD users and an equal number of matched nonusers within the overall DKD patient population. For advanced DKD patients, the cohort comprised 1,422 PRCHMDKD users and an equivalent number of matched nonusers.

**Results:**

PRCHMDKD use in overall and advanced, respectively, DKD patients was associated with time-dependent reductions in adjusted hazard ratios for ESRD (0.66; 95% CI, 0.61–0.70 vs. 0.81; 0.65–0.99), all-cause mortality (0.48; 0.47–0.49 vs. 0.59; 0.50–0.70), and cardiovascular mortality (0.50; 0.48–0.53 vs. 0.61; 0.45–0.82). Significant differences in RMST were observed in overall and advanced, respectively, DKD patients, favoring PRCHMDKD use: 0.31 years (95% CI, 0.24–0.38) vs. 0.61 years (0.13–1.10) for ESRD, 2.71 years (2.60–2.82) vs. 1.50 years (1.03–1.98) for all-cause mortality, and 1.18 years (1.09–1.28) vs. 0.59 years (0.22–0.95) for cardiovascular mortality. Additionally, hyperkalemia risk did not increase. These findings remained consistent despite multiple sensitivity analyses. Notably, the cumulative effects of utilizing at least four or five classes and multiple botanical drugs from the sixteen PRCHMDKD provided enhanced renoprotection for patients with both overall and advanced DKD. This suggests that there is involvement of multiple targets within the oxidative stress pathways associated with DKD.

**Conclusion:**

This real-world study suggests that using these sixteen PRCHMDKD provides time-dependent cardiorenal and survival benefits while ensuring safety for DKD patients.

## 1 Introduction

Over the last few decades, there has been a consistent rise in the prevalence and incidence of diabetes, along with an elevated risk of cardiovascular diseases, mortality, and the development of diabetic kidney disease (DKD) that leads to end-stage renal disease (ESRD), all of which have placed significant financial burdens on the nation (Koye et al., 2018). Although renin-angiotensin system inhibitors and sodium-glucose cotransporter two inhibitors have emerged as the primary treatments for DKD, it has proven challenging to effectively halt the ongoing decline in kidney function, which ultimately leads to ESRD and its associated cardiovascular complications (Chen D. Q. et al., 2022).

A causal link exists between oxidative stress and inflammation in the progression of renal and cardiovascular complications associated with diabetes (Jha et al., 2018). Furthermore, research has shown that oxidative stress mechanisms can potentially be targeted for the treatment of chronic kidney disease (CKD) (Dobrek, 2022). Chinese herbal medicines (CHMs), including active metabolites, extracts, and formulations, in conjunction with Western medicine, offer multiple therapeutic avenues for addressing DKD. These approaches work synergistically to mitigate oxidative stress and inflammation (Chen D. Q. et al., 2022), ultimately slowing down DKD progression (Jiang et al., 2021). In Taiwan, National Health Insurance (NHI) strictly regulates and covers CHM services (Chen et al., 2022b). CHMs containing aristolochic acid were excluded from Taiwan’s universal NHI program starting in 2003 (Chen et al., 2015; Chen et al., 2023). All prescribed CHMs eligible for NHI coverage in Taiwan are known as scientific Chinese medicine (Lu et al., 2005; Chen et al., 2015). These botanical drugs are based on the Taiwan herbal pharmacopeia (2022) and refer to pre-prepared, concentrated Chinese medicines (Lu et al., 2005), primarily available as granules or powders (Chen et al., 2015). They are derived from raw materials that have undergone modern processes, including grinding, boiling, filtering, concentrating, drying, and blending with an excipient (Lu et al., 2005). The value of treating patients with CKD using prescribed CHM in Taiwan has gained attention because of emerging evidence highlighting the potential renal and survival benefits of prescribed CHM for both overall (Lin et al., 2015) and advanced (Chen et al., 2022b) CKD, and advanced DKD (Guo et al., 2021). This attention has been further fueled by the introduction of a traditional Chinese medicine-enhanced CKD outpatient care program supported by Taiwan’s National Health Insurance (NHI) administration (Chen et al., 2023).

Sixteen potentially renoprotective CHMs for diabetic kidney disease (PRCHMDKD) were identified based on evidence from systematic meta-analyses, review articles, and randomized controlled trials (outlined in detail in the eMethod section of the Supplementary Material) and categorized into five distinct classes (Cao, 2000; Jin et al., 2011) (detailed in Supplementary Table S1). The crucial renoprotective mechanisms of these sixteen PRCHMDKD include the modulation of glucose/lipid metabolism, antioxidative, anti-inflammatory, anti-fibrotic activities, and the safeguarding of podocytes (Supplementary Table S2), with antioxidative action being a common property. These sixteen PRCHMDKD include clearing heat (*Rehmannia glutinosa* (Gaertn.) DC [Orobanchaceae; Radix Rehmanniae] (Sheng-Di-Huang), *Anemarrhena asphodeloides* Bunge [Asparagaceae; Rhizoma Anemarrhenae] (Zhi-Mu), *Salvia miltiorrhiza* Bunge [Lamiaceae; Radix et Rhizoma Salviae Miltiorrhizae] (Dan-Shan), *Rheum palmatum* L [Polygonaceae; Radix et Rhizoma Rhei] (Da-Huang), and *Coptis chinensis* Franch [Ranunculaceae; Rhizoma Coptidis] (Huang-Lian)), nourishing yin (*Paeonia lactiflora* Pall [Paeoniaceae; Radix Paeoniae Alba] (Bai-Shao), *Cornus officinalis* Sieb. et Zucc [Cornaceae; Fructus Corni] (Shan-Zhu-Yu), *Pueraria lobata* (Willd.) Ohwi [Fabaceae; Radix Puerariae] (Ge-Gen), and Liu-Wei-Di-Huang-Wan), dampness dispelling (*Ligusticum striatum* DC [Apiaceae; Rhizoma Chuanxiong] (Chuan-Xiong), *Plantago asiatica* L [Plantaginaceae; Herba Plantaginis] (Che-Qian-Cao), Shen-Qi-Wan, and Wu-Ling-San), tonifying qi (*Astragalus membranaceus* (Fisch.) Bunge [Fabaceae; Radix Astragali] (Huang-Qi) and *Panax notoginseng* (Burkill) F.H. Chen [Araliaceae; Radix Notoginseng] (San-Qi)), and harmonizing formulas (Xiao-Chai-Hu-Tang) (Lu et al., 2019; Tang G. et al., 2021; Chen D. Q. et al., 2022). Acknowledging localized tissue oxidative stress as a prominent culprit for the development of DKD (Forbes et al., 2008) and recognizing the importance of combined therapy of CHM for the effective treatment of DKD, aiming to target multiple pathways associated with oxidative stress (Chen D. Q. et al., 2022), we hypothesize that the beneficial effects of these sixteen PRCHMDKD, as documented in the treatment of DKD and characterized by a shared antioxidative action, could be elucidated through an analysis of a national health insurance database. Our objective is to investigate the time-response relationship of these sixteen PRCHMDKD and explore the additive effects of one to sixteen and one to five classes of PRCHMDKD on cardiorenal and survival outcomes, which currently remain unresolved. Moreover, herbal remedies are considered potential triggers of hyperkalemia in patients with CKD, particularly in those with diabetes (Goia-Nishide et al., 2022) and advanced CKD (Chen et al., 2022b). The association of sixteen PRCHMDKD with hyperkalemia risk remains undetermined. Because these sixteen PRCHMDKD were eligible for NHI coverage, we analyzed national claims data, which include a highly representative sample, to fill this knowledge gap in patients with overall and advanced DKD and establish a foundation for future research in this area.

## 2 Materials and methods

This study followed the “Consensus statement on the Phytochemical Characterisation of Medicinal Plant extracts” (ConPhyMP) guidelines, focusing exclusively on “type A” extracts as recognized in the national pharmacopeia and possessing appropriate licensure (Heinrich et al., 2022).

### 2.1 Data source and study design

The cohort study utilized a retrospective design and analyzed claims data from 2000 to 2017 of Taiwan’s 2005 Longitudinal Generation Tracking Database (LGTD2005) (Chen et al., 2022b), covering the period from 2000 to 2017. This database comprises the data of two million de-identified individuals randomly selected from the entire pool of beneficiaries of Taiwan’s NHI program. Therefore, this study did not require informed consent and was exempt from full review by the Institutional Review Board of the Dalin Tzu Chi Hospital (B10804001). The specifics within LGTD2005 and the NHI program were elucidated in our earlier research (Chen et al., 2015; Lin et al., 2015; Chen et al., 2022b; Chen et al., 2022c; Chen et al., 2023). Briefly, LGTD2005 is a comprehensive medical database that provides detailed information on medications and herbal treatments, excluding laboratory and lifestyle data. It utilizes the ICD-9-CM and ICD-10-CM (starting in 2016) diagnostic codes for disease definitions (Chen et al., 2022b; Chen et al., 2023).

### 2.2 Study population (patients with overall and advanced DKD) exposed to sixteen potentially renoprotective Chinese herbal medicines for diabetic kidney disease (PRCHMDKD)

**FIGURE 1:**
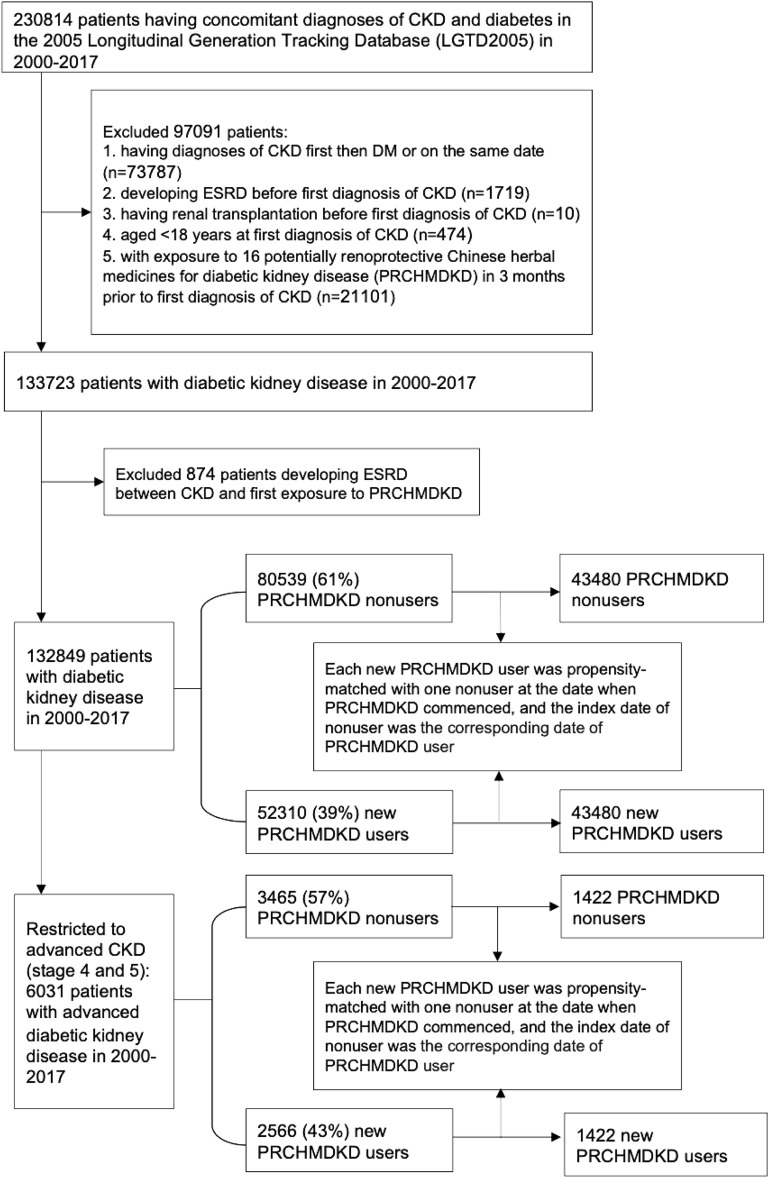
Flowchart for selecting patients with overall and advanced diabetic kidney disease.

The study cohort identified 230,814 patients with concomitant diagnoses of CKD and diabetes between 1 January 2000, to 31 December 2017 (Figure 1). We excluded patients who either had a diagnosis of first CKD (defined by ICD 9/10-CM codes) followed by diabetes (defined by ICD 9/10-CM codes or antihyperglycemic drugs) or received diagnoses of both conditions on the same date, developed ESRD, or underwent renal transplantation prior to the first diagnosis of CKD. Additionally, patients aged <18 years at the time of their first CKD diagnosis and those who had been exposed to sixteen PRCHMDKD in the 3 months preceding their first diagnosis of CKD were also excluded. Therefore, we enrolled 133,723 patients with overall DKD from 2000–2017.

DKD patients were categorized as PRCHMDKD users if they received at least one of these 16 PRCHMDKD (Supplementary Table S1) after CKD diagnosis during the study period. Those who never used any of these 16 PRCHMDKD after CKD diagnosis were defined as nonusers. This approach is consistent with the user and nonuser definitions utilized in our prior research (Chen et al., 2024). Based on the exposure to PRCHMDKD after the onset of CKD and excluding patients who developed ESRD between CKD and their first exposure to PRCHMDKD, we identified 132,849 patients with overall DKD in the years 2000–2017, including 52,310 (39%) new PRCHMDKD users and 80,539 (61%) nonusers. Advanced CKD was identified by applying ICD-9-CM codes for CKD in conjunction with erythropoiesis-stimulating agent use, ICD-10-CM codes for CKD stage 5 with or without erythropoiesis-stimulating agent use, and ICD-10-CM codes for CKD stage 4 (Chen et al., 2022b). Hence, 6,031 patients with advanced DKD (stages 4 and 5) were identified, including 2,566 (43%) new PRCHMDKD users and 3,465 (57%) nonusers.

### 2.3 Covariate assessments

Several variables were evaluated, including age, sex, Charlson comorbidity index (a measure of overall disease burden in patients) (Chen et al., 2022b; Chen et al., 2023), frequency of medical visits (to reduce detection bias) (Chen et al., 2022b; Chen et al., 2022c; Chen et al., 2023), baseline comorbidities such as hypertension (defined by ICD 9/10-CM codes or antihypertensives), coronary heart disease (defined by ICD 9/10-CM codes), hyperlipidemia (defined by ICD 9/10-CM codes or antilipidemic drugs), and chronic liver disease (defined by ICD 9/10-CM codes) (Chen et al., 2022b). Additionally, the use of three potentially confounding medications (non-steroidal anti-inflammatory drugs, anti-hyperglycemic drugs, and angiotensin-converting enzyme inhibitors/angiotensin Ⅱ receptor blockers) within the 1-year period preceding the index date were also assessed.

### 2.4 Propensity score matching

Propensity score matching was conducted using the nearest-neighbor approach without replacement, employing a caliper value of 0.0001 to maintain precision throughout the matching process (Chen et al., 2022b). The propensity score was calculated using the logistic regression that was built on all covariates (age per year, sex, comorbidities, number of medical visits, Charlson comorbidity index, and confounding drugs) to adjust for the baseline differences between PRCHMDKD users and nonusers. For patients with overall DKD, a propensity score-matched nonuser was selected for each PRCHMDKD user. To mitigate immortal bias (Chen et al., 2015; Chen et al., 2022b; Chen et al., 2023), each matched nonuser was ensured to be alive at the initiation of PRCHMDKD use. The index date for PRCHMDKD users was defined as the exact day on which PRCHMDKD therapy was commenced, ensuring that PRCHMDKD user survived from the onset of CKD until this index date. Similarly, for nonusers, the index date was synchronized with the corresponding day of the PRCHMDKD commencement by the user (Chen et al., 2023; Wakabayashi et al., 2023; Chen et al., 2024). The same propensity score matching method was applied to patients with advanced DKD.

### 2.5 Study endpoints and follow-up

The primary study endpoints encompassed ESRD, all-cause mortality, and cardiovascular mortality. Cardiovascular mortality was defined as death attributed to the principal diagnoses, including coronary heart disease, stroke, peripheral vascular disease, and heart failure identified by ICD 9/10-CM codes (Yu et al., 2022). Patients were observed from their index date until the onset of ESRD or instances of censoring such as mortality or the conclusion of the study (31 December 2017), whichever transpired first. The definition of ESRD relied on the possession of a catastrophic illness certificate for long-term dialysis (Chen et al., 2022b; Chen et al., 2022c; Chen et al., 2023). Mortality was ascertained based on patient withdrawal from the NHI program (Chen et al., 2022b; Chen et al., 2022c; Chen et al., 2023). During the analysis of mortality outcomes, patients were continuously tracked until their death occurred, and instances of ESRD were not considered censoring points if they had occurred earlier (Huang et al., 2022). The secondary study endpoint was the occurrence of hyperkalemia during the study period. Hyperkalemia was identified through outpatient and inpatient records throughout the study period. This identification was based on the presence of ICD-9/10-CM codes for hyperkalemia, use of potassium-lowering agents, or procedure codes indicating immediate hemodialysis in conjunction with ICD-9/10-CM codes for hyperkalemia (Chen et al., 2022b).

### 2.6 Time-response relationship between sixteen PRCHMDKD and study outcomes

To examine the relationship between the duration of PRCHMDKD exposure and the primary study endpoints over time, we analyzed the prescription duration of PRCHMDKD in patients with both overall and advanced DKD. This analysis was conducted on participants with follow-up periods extending beyond 1 year. The duration was measured in cumulative days of PRCHMDKD usage and divided into three categories (1–30 days, 31–120 days, and ≥121 days), with nonuse as the reference category.

### 2.7 Additional analyses

We classified sixteen PRCHMDKD into five classes: clearing heat, nourishing yin, dampness dispelling, tonifying qi, and harmonizing formulas (Jin et al., 2011; Lin et al., 2015; Huang et al., 2022; detailed in Supplementary Table S1). We evaluated the association of the additive effects of one to five classes of PRCHMDKD and one to sixteen PRCHMDKD on ESRD risk. We also performed the subgroup analysis in terms of the five classes for survival outcome in patients with overall and advanced DKD.

### 2.8 Statistical analyses

We assessed disparities in baseline characteristics between PRCHMDKD users and nonusers using a standardized mean difference approach. A standardized mean difference value of <0.1 indicated a minimal distinction between the two groups following propensity score matching (Chen et al., 2022b). For both groups, we calculated the incidence rates per 100 person-years for the study outcomes and verified the assumption of proportional hazards by creating a graph of log (-log (survival)) against the log of survival time, which showed no violations. The primary study endpoints were assessed using the Cox proportional hazard model and complementary restricted mean survival time (RMST) analyses. Adjusted hazard ratios (aHRs) and the associated 95% confidence intervals (CIs) were computed by comparing PRCHMDKD users with nonusers. These estimations considered all covariates listed in Table 1 and accounted for competing mortality when evaluating the risk of ESRD (Chen et al., 2015; Chen et al., 2023). We used modified Kaplan-Meier and Gray’s methods (Chen et al., 2015) to calculate and compare the cumulative incidence of ESRD in data with competing risk, and employed the Kaplan-Meier method to estimate the cumulative incidence of overall mortality. Differences in the complete time-to-event distributions between the study groups in patients with overall and advanced DKD were assessed utilizing a modified log-rank test for ESRD and a log-rank test for overall mortality.

**TABLE 1:** Baseline characteristics of study cohorts in patients with diabetic kidney disease (DKD) by use of 16 potentially renoprotective Chinese herbal medicines for diabetic kidney disease (PRCHMDKD).

	Propensity-matched overall DKD patients (n = 86,960)			Propensity-matched advanced DKD patients (n = 2,844)		
	User (n = 43,480, %)	Nonuser (n = 43,480, %)	SMD/*p*-value	User (n = 1,422, %)	Nonuser (n = 1,422, %)	SMD/*p*-value
Distribution of CKD stage						
1–3 (mild-to-moderate)	41,212 (94.8)	41,623 (95.7)	—	—	—	—
4–5 (advanced)	2,268 (5.2)	1857 (4.3)	—	—	—	—
4	1713 (3.9)	1,315 (3.0)	—	1,074 (75.5)	1,036 (72.9)	—
5	555 (1.3)	542 (1.3)	—	348 (24.5)	386 (27.1)	—
Variables						
Sex			0.012/0.09			0.014/0.71
Men	21,767 (50.1)	22,018 (50.6)		792 (55.7)	782 (55.0)	
Women	21,713 (49.9)	21,462 (49.4)		630 (44.3)	640 (45.0)	
Age (year)			0.029/0.57			0.031/0.99
18–50	8,697 (20.0)	8,755 (20.1)		167 (11.7)	162 (11.4)	
51–60	11,159 (25.7)	10,956 (25.2)		334 (23.5)	330 (23.2)	
61–70	12,230 (28.1)	12,241 (28.2)		444 (31.2)	456 (32.1)	
71–80	9,016 (20.7)	9,138 (21.0)		368 (25.9)	366 (25.7)	
>81	2,378 (5.5)	2,390 (5.5)		109 (7.7)	108 (7.6)	
Mean (SD)	61.1 (13.3)	61.5 (13.4)		64.4 (11.8)	64.7 (11.8)	
Comorbidities (yes)						
Hypertension	30,249 (69.6)	30,464 (70.1)	0.011/0.11	1,198 (84.3)	1,218 (85.7)	0.039/0.29
Coronary heart disease	7,788 (17.9)	7,614 (17.5)	0.010/0.12	313 (22.0)	325 (22.9)	0.020/0.59
Hyperlipidemia	19,849 (45.7)	19,640 (45.2)	0.010/0.15	767 (53.9)	774 (54.4)	0.010/0.79
Chronic liver disease	9,259 (21.3)	9,024 (20.8)	0.013/0.05	225 (15.8)	205 (14.4)	0.039/0.30
Charlson comorbidity index			0.007/0.29			0.036/0.66
≤1	17,061 (39.3)	16,850 (38.8)		225 (15.8)	240 (16.9)	
2	12,434 (28.6)	12,669 (29.1)		459 (32.3)	474 (33.3)	
3	7,889 (18.1)	7,914 (18.2)		386 (27.1)	380 (26.7)	
≥4	6,096 (14.0)	6,047 (13.9)		352 (24.8)	328 (23.1)	
Mean (±SD)	2 (1.4)	1.99 (1.5)		2.73 (1.4)	2.68 (1.4)	
No. Of medical visits			0.022/0.48			0.018/0.98
≤12	12,391 (28.5)	12,249 (28.2)		316 (22.2)	316 (22.2)	
12–24	14,206 (32.7)	14,342 (33.0)		450 (31.7)	455 (32.0)	
>24	16,883 (38.8)	16,889 (38.8)		656 (46.1)	651 (45.8)	
Mean (SD)	24.32 (18.7)	23.92 (18.2)		26.92 (19.1)	26.58 (18.4)	
Confounding drugs (yes)						
NSAID	33,729 (77.6)	33,881 (77.9)	0.008/0.22	1,105 (77.7)	1,111 (78.1)	0.010/0.79
ACEI/ARB	18,906 (43.5)	18,970 (43.6)	0.003/0.66	951 (66.9)	953 (67.0)	0.003/0.94
Anti-hyperglycemic drugs	28,041 (64.5)	27,834 (64.0)	0.010/0.14	1,173 (82.5)	1,180 (83.0)	0.013/0.73

Categorical variables given as number (percentage); continuous variable, as mean ± standard deviation (SD)

Abbreviations: ACEI/ARB, angiotensin-converting-enzyme inhibitor/angiotensin II receptor blocker; SMD, standardized mean difference; NSAID, nonsteroid anti-inflammatory drugs.

RMST, the area under the survival curve between zero and a specified (restricted) time, has been advocated as an alternative measure to complement traditional Cox analysis. The RMST difference allows the quantification of the postponement of an outcome during a specified (restricted) interval and corresponds to the difference between the areas under the two survival curves for the intervention and control groups. Therefore, the RMST difference can be used to assess whether a benefit is clinically significant as opposed to the relative treatment effects provided by HRs (Perego et al., 2020). We evaluated the differences in the RMST by comparing the areas under the survival curves between PRCHMDKD users and nonusers. A positive RMST difference favored PRCHMDKD treatment and signified a mean postponement in reaching the primary study endpoints between the two groups. We further evaluated the 17-year RMST difference, along with its corresponding 95% CI, for the primary study endpoints between PRCHMDKD users and nonusers. This comparison complements the insights derived from aHR estimates and provides a comprehensive understanding of the findings.

Poisson regression was used to estimate the adjusted incident rate ratio of hyperkalemia associated with prescribed CHM throughout the study. Recurrent episodes of hyperkalemia were treated as distinct events if they occurred at least 28 days apart and were considered as prolonged events if they occurred within a span of less than 28 days (Chen et al., 2022b; Chen et al., 2023).

Statistical analyses were conducted using SAS software (version 9.4; SAS Institute, Inc., Cary, N.C., United States). Statistical significance was established when the 95% CI for aHRs did not encompass one or when the 95% CI for the difference in RMST did not encompass 0. A two-tailed *p*-value below 0.05 was considered statistically significant.

### 2.9 Sensitivity analyses

Seven sensitivity analyses were performed on patients with overall and advanced DKD to enhance the robustness of the key findings. First, the PRCHMDKD-usage group was redefined based on cumulative usage days as >30 and >60 days. Second, we revisited the risk assessment for study outcomes by excluding patients with CKD who either died or progressed to ESRD within 30 and 60 days following the index date. Third, we performed subgroup analyses according to the baseline characteristics. Fourth, we incorporated variables (such as hypertension, hyperlipidemia, and chronic liver disease) that could potentially influence the utilization of PRCHMDKD between overall DKD/advanced DKD and the PRCHMDKD index date into the regression model. Fifth, we considered two confounding drugs (sodium–glucose cotransporter two inhibitors and glucagon-like peptide-1 agonists), which are known to positively influence cardiorenal and mortality outcomes, into the regression models. Sixth, we incorporated two covariates (stroke and peripheral arterial occlusion disease) into the regression model. Seventh, to mitigate the potential antioxidative influence of alternative CHMs apart from PRCHMDKD, we defined nonusers as patients with DKD/advanced DKD who had never utilized PRCHMDKD or any other CHMs. Subsequently, we reassessed the associations of the three outcomes between PRCHMDKD users and nonusers.

## 3 Results

### 3.1 Patient characteristics

After performing propensity score matching, a suitable balance was attained across all baseline characteristics between 43,480 users and 43,480 nonusers in patients with overall DKD, as well as between 1,422 users and 1,422 nonusers in patients with advanced DKD (Table 1). The distribution of CKD stage in propensity-matched overall and advanced DKD patients was detailed in the eResult section of the Supplementary Material. Consequently, this resulted in acceptable discrimination between matched cohorts, with a c-index of 0.67 and 0.65, along with Hosmer–Lemeshow test *p*-value of >0.05, for patients with overall and advanced DKD, respectively.

### 3.2 Association between sixteen PRCHMDKD and study outcomes

In patients with overall DKD, 3,469 (16.6%) progressed to ESRD, whereas 19,523 (22.5%) died prior to ESRD progression. Additionally, the number for all-cause and cardiovascular deaths was 21,588 (24.8%) and 6,997 (8%), respectively. The incidence rates of ESRD (0.69 vs. 1.18/100 patient-years), all-cause mortality (3.67 vs. 7.82/100 patient-years), and cardiovascular mortality (0.12 vs. 0.25/100 patient-years) were significantly lower (*p* < 0.0001) in PRCHMDKD users than in nonusers (Table 2). In overall DKD patients, the median follow-up time (Supplementary Table S3) for ESRD, all-cause mortality, and cardiovascular mortality was 5.14, 5.44, and 5.44 years among PRCHMDKD users, compared to 1.87, 2.01, and 2.01 years among nonusers. In advanced DKD patients, the median follow-up time for ESRD, all-cause mortality, and cardiovascular mortality was 6.24, 7.20, and 7.20 years among PRCHMDKD users, compared to 3.88, 4.54, and 4.54 years among nonusers.

**TABLE 2:** 17-year restricted mean survival time (RMST) and Cox proportional hazards model analyses on study outcomes among propensity score–matched PRCHMDKD users and nonusers in patients with overall and advanced DKD.

	Events (%)		Incidence rate/100 person-years		RMST (95% CI), year		RMST difference (year, 95% CI)	Adjusted HR (95% CI)
	User	Nonuser	User	Nonuser	User	Nonuser		
Overall DKD patients								
ESRD	1856 (4.3)	1,613 (3.7)	0.69	1.18	16.08 (16.04, 16.12)	15.77 (15.7, 15.83)	0.31 (0.24, 0.38)	0.66 (0.61, 0.70)
All death	10,230 (23.5)	11,358 (26.1)	3.67	7.82	12.56 (12.5, 12.63)	9.57 (9.47, 9.67)	2.71 (2.60, 2.82)	0.48 (0.47, 0.49)
CV death	3,386 (7.8)	3,611 (8.3)	0.12	0.25	15.3 (15.24, 15.35)	13.93 (13.82, 14.03)	1.18 (1.09, 1.28)	0.50 (0.48, 0.53)
Advanced DKD patients								
ESRD	170 (12.0)	166 (11.7)	1.70	2.31	14.78 (14.47, 15.09)	14.16 (13.76, 14.57)	0.61 (0.13, 1.10)	0.81 (0.65, 0.99)
All death	248 (17.4)	282 (19.8)	2.27	3.51	14.16 (13.86, 14.46)	12.57 (12.17, 12.97)	1.50 (1.03, 1.98)	0.59 (0.50, 0.70)
CV death	83 (5.8)	93 (6.5)	0.08	0.12	15.93 (15.71, 16.15)	15.31 (14.98, 15.64)	0.59 (0.22, 0.95)	0.61 (0.45, 0.82)

Abbreviations: PRCHMDKD, potentially renoprotective Chinese herbal medicines for diabetic kidney disease; HR, hazard ratio; CI, confidence interval; ESRD, end-stage renal disease; CV, cardiovascular. Adjusted for all covariates (age per year, sex, comorbidities, number of medical visits, Charlson comorbidity index, and confounding drugs) and competing mortality for ESRD.

The 17-year cumulative incidences of ESRD (*p* < 0.0001 and *p* = 0.047, respectively) in the presence of competing mortality and overall mortality (*p* < 0.0001 and *p* < 0.0001, respectively) were markedly lower for PRCHMDKD users compared to nonusers in patients with overall (Supplementary Figure S1) and advanced (Supplementary Figure S2) DKD, respectively. Upon accounting for all covariates, PRCHMDKD use in patients with overall DKD was significantly associated with reduced risks of ESRD (aHR: 0.66; 95% CI: 0.61–0.70), all-cause mortality (aHR: 0.48; 95% CI: 0.4–0.49) and cardiovascular mortality (aHR: 0.50; 95% CI: 0.48–0.53). Over a span of 17 years, PRCHMDKD use, as opposed to nonuse, was associated with a delay of 0.31 (95% CI: 0.24–0.38), 2.71 (95% CI: 2.60–2.82), and 1.18 years (95% CI: 1.09–1.28) in the occurrence of ESRD, all-cause mortality, and cardiovascular mortality, respectively. Given the type I error α of 0.05, the event rate per year of 0.078 for nonuser group, median follow-up of 4.88 years, censoring rate of 0.92, and user-to-nonuser ratio of 1:1, it takes 25,402 in both user and nonuser groups to have a power (1-β) of 90% to detect a 10% change in HR. Our sample size of 43,480 in each group with aHR of 0.47 suggests a test power of greater than 90%.

Among patients with advanced DKD, 336 (11.8%) progressed to ESRD, while 439 (15.4%) died prior to ESRD progression. Additionally, the number for all-cause and cardiovascular deaths was 530 (18.6%) and 176 (6.2%), respectively. The incidence rates of ESRD (1.70 vs. 2.31/100 patient-years), all-cause mortality (2.27 vs. 3.51/100 patient-years), and cardiovascular mortality (0.08 vs. 0.12/100 patient-years) were significantly lower (*p* < 0.0001) in PRCHMDKD users than in nonusers. Upon accounting for all covariates, PRCHMDKD use in patients with advanced DKD was significantly associated with reduced risks of ESRD (aHR: 0.81; 95% CI: 0.65–0.99), all-cause mortality (aHR: 0.59; 95% CI: 0.50–0.70) and cardiovascular mortality (aHR: 0.61; 95% CI: 0.45–0.82). Over a span of 17 years, PRCHMDKD use, as opposed to nonuse, was associated with a delay of 0.61 (95% CI: 0.13–1.10), 1.50 years (95% CI: 1.03–1.98), and 0.59 (95% CI: 0.22–0.95) in the occurrence of ESRD, all-cause mortality, and cardiovascular mortality, respectively.

### 3.3 Time-response relationship between sixteen PRCHMDKD and study outcomes

When comparing to nonuse as the reference, we observed a time-response relationship of PRCHMDKD use with the risks of ESRD and all-cause and cardiovascular mortality in patients with overall and advanced DKD busing both the Cox model and RMST analyses (Table 3). These relationships were apparent when considering cumulative exposure to PRCHMDKD in days categorized into three tiers (1–30, 31–120, and ≥121 days) during a minimum follow-up period of 1 year.

**TABLE 3:** Cumulative exposure duration of 16 PRCHMDKD use and risk of study outcomes at least >1 year of follow-up.

Cumulative exposure In days	ESRD			All death			CV death		
	Event (%)	RMSTd (y) (95% CI)	aHR (95% CI)	Event (%)	RMSTd (y) (95% CI)	aHR (95% CI)	Event (%)	RMSTd (y) (95% CI)	aHR (95% CI)
Overall DKD patients									
Nonuse	955 (3.3%)	0 (References)	1 (References)	7,922 (27.1%)	0 (References)	1 (References)	2,545 (8.7%)	0 (References)	1 (References)
Use	1,473 (3.9%)	0.17 (0.11, 0.24)	0.74 (0.68, 0.80)	9,103 (23.7%)	2.05 (1.94, 2.16)	0.53 (0.52, 0.55)	3,040 (7.9%)	0.87 (0.78, 0.96)	0.56 (0.53, 0.59)
1–30	488 (4.4%)	−0.08 (−0.18, 0.01)	1.01 (0.91, 1.13)	3,078 (27.4%)	0.58 (0.43, 0.73)	0.77 (0.74, 0.80)	1,029 (9.2%)	0.21 (0.07, 0.34)	0.78 (0.73, 0.84)
31–120	476 (4.1%)	0.09 (−0.01, 0.19)	0.80 (0.71, 0.89)	2,902 (24.8%)	1.66 (1.51, 1.82)	0.57 (0.55, 0.60)	1,006 (8.6%)	0.61 (0.48, 0.75)	0.61 (0.57, 0.66)
≥121	509 (3.3%)	0.43 (0.34, 0.51)	0.55 (0.49, 0.61)	3,123 (20.2%)	3.42 (3.28, 3.56)	0.39 (0.37, 0.40)	1,005 (6.5%)	1.54 (1.43, 1.66)	0.40 (0.37, 0.43)
Advanced DKD patients									
Nonuse	131 (11%)	0 (References)	1 (References)	244 (20.0%)	0 (References)	1 (References)	77 (6.3%)	0 (References)	1 (References)
Use	145 (11.3%)	0.50 (0.04, 0.97)	0.76 (0.60, 0.97)	230 (17.6%)	1.35 (0.89, 1.81)	0.61 (0.51, 0.73)	76 (5.8%)	0.49 (0.14, 0.85)	0.64 (0.47, 0.89)
1–30	45 (12.7%)	−0.20 (−0.91, 0.50)	1.08 (0.76, 1.52)	77 (21.0%)	0.13 (−0.57, 0.82)	0.96 (0.74, 1.24)	25 (6.8%)	−0.01 (−0.57, 0.54)	0.99 (0.63, 1.56)
31–120	42 (11.6%)	0.36 (−0.36, 1.07)	0.79 (0.56, 1.12)	63 (17.1%)	1.24 (0.56, 1.92)	0.64 (0.48, 0.84	26 (7.1%)	0.22 (−0.34, 0.79)	0.80 (0.51, 1.26)
≥121	58 (10.1%)	1.04 (0.43, 1.64)	0.61 (0.44, 0.83)	90 (15.7%)	2.20 (1.61, 2.80)	0.45 (0.35, 0.57)	25 (4.3%)	1.00 (0.57, 1.42)	0.41 (0.26, 0.64)

Abbreviations: the same as Tables 1, 2

Adjusted for all covariates (age per year, sex, comorbidities, number of medical visits, Charlson comorbidity index, confounding drugs) and competing mortality for ESRD.

### 3.4 Additive effects of one to five classes of sixteen PRCHMDKD on ESRD outcome in patients with overall and advanced DKD

Among patients with overall DKD, top renoprotection occurred with the use of all five classes (aHR: 0.20; 95% CI: 0.15–0.27), followed by four classes (clearing heat, nourishing yin, and dampness dispelling, along with harmonizing formulas [aHR: 0.45; 95% CI: 0.27–0.73] or tonifying qi [aHR: 0.45; 95% CI: 0.40–0.51]), and three classes (clearing heat, nourishing yin, and dampness dispelling) (aHR: 0.69; 95% CI: 0.59–0.81), which remained consistent in RMST analysis (Table 4). Among patients with advanced DKD, the top renoprotection occurred with the use of four classes (clearing heat, nourishing yin, dampness dispelling, and tonifying qi) (aHR: 0.52; 95% CI: 0.35–0.76).

**TABLE 4:** Additive effect of 16 PRCHMDKD by five classes on end-stage renal disease (ESRD) outcome in patients with overall and advanced DKD.

Clearing heat	Nourishing yin	Dampness dispelling	Tonifying qi	Harmonizing formulas	ESRD			
					Event	N	RMST difference (year, 95% CI)	aHR (95% CI)
Overall DKD patients								
V	V	V	V	V	79	3,775	0.85 (0.68, 1.01)	0.20 (0.15, 0.27)
V	V	V		V	31	840	0.60 (0.14, 1.06)	0.45 (0.27, 0.73)
V	V	V	V		499	12,378	0.53 (0.41, 0.64)	0.45 (0.40, 0.51)
V	V	V			370	8,362	0.28 (0.11, 0.45)	0.69 (0.59, 0.81)
Advanced DKD patients								
V	V	V	V		44	419	1.33 (0.52, 2.14)	0.52 (0.35, 0.76)

Abbreviations: the same as Tables 1-3

Adjusted for all covariates (age per year, sex, comorbidities, number of medical visits, Charlson comorbidity index, and confounding drugs) and competing risk for ESRD.

### 3.5 Additive effect of one to sixteen PRCHMDKD on ESRD outcome in patients with overall DKD

Table 5 shows the top seven renoprotective botanical drugs among the sixteen PRCHMDKD, which correspond to the use of five classes (clearing heat, nourishing yin, dampness dispelling, harmonizing formulas, and tonifying qi) and four classes (clearing heat, nourishing yin, dampness dispelling, and tonifying qi), as shown in Table 4. The most effective renoprotective compositions were Liu-Wei-Di-Huang-Wan, *C. chinensis* (Huang-Lian), *R. glutinosa* (Sheng-Di-Huang), *P. lactiflora* (Bai-Shao), *C. officinalis* (Shan-Zhu-Yu), *P. lobata* (Ge-Gen), *A. asphodeloide*s (Zhi-Mu), *L. striatum* (Chuan-Xiong), *A. membranaceus* (Huang-Qi), *S. miltiorrhiza* (Dan-Shan), and *R. palmatum* (Da-Huang), along with Xiao-Chai-Hu-Tang indicating a delay of 1.28 years (95% CI: 0.64–1.93) or Shen-Qi-Wan and Wu-Ling-San indicating a delay of 1.14 years (95% CI: 0.39–1.90).

**TABLE 5:** Additive effect of one to sixteen items of PRCHMDKD on end-stage renal disease (ESRD) outcome in patients with overall DKD.

Item 1	Item 2	Item 3	Item 4	Item 5	Item 6	Item 7	Item 8	Item 9	Item 10	Item 11	Item 12	Item 13	Item 14	Item 15	Item 16	ESRD		
																Event/number	RMST difference (year, 95% CI)	Adjusted HR (95% CI)
	V			V		V	V	V		V	V	V	V	V		9/272	0.94 (0.26, 1.61)	0.14 (0.05, 0.38)
	V			V		V	V	V		V	V	V	V	V	V	5/254	0.84 (0.27, 1.42)	0.16 (0.05, 0.53)
	V		V	V		V	V	V		V	V	V	V	V	V	1/202	1.28 (0.64, 1.93)	0.04 (0, 0.30)
V	V		V	V		V	V	V		V	V	V	V	V	V	1/187	0.85 (0.29, 1.41)	0.06 (0.01, 0.57)
				V		V	V			V	V	V	V	V		2/150	0.85 (0.10, 1.59)	0.08 (0.01, 0.59)
V	V	V		V		V	V	V		V	V	V	V	V	V	3/144	1.14 (0.39, 1.90)	0.03 (0, 0.25)
	V		V	V		V	V	V		V	V	V	V	V		2/140	0.92 (0.13, 1.72)	0.10 (0.01, 0.64)

Abbreviation: the same as Tables 1-4; Item 1, Shen-Qi-Wan; Item 2, Liu-Wei-Di-Huang-Wan; Item 3, Wu-Ling-San; Item 4, Xiao-Chai-Hu-Tang; Item 5, Huang-Lian; Item 6, Che-Qian-Cao; Item 7, Sheng-Di-Huang; Item 8, Bai-Shao; Item 9, Shan-Zhu-Yu; Item 10, San-Qi; Item 11, Ge-Gen; Item 12, Zhi-Mu; Item 13, Chuan-Xiong; Item 14, Huang-Qi; Item 15, Dan-Shan; Item 16, Da-Huang.

### 3.6 Subgroup analysis in terms of five classes of sixteen PRCHMDKD for survival outcome

In patients with overall and advanced DKD, all five classes of sixteen PRCHMDKD were significantly associated with a lower risk of overall mortality (Supplementary Table S4). Notably, harmonizing formulas provided the most benefit for survival in patients with overall and advanced DKD.

### 3.7 Hyperkalemia risk associated with sixteen PRCHMDKD

No association between increased hyperkalemia risk and PRCHMDKD use was observed in patients with overall (aHR, 0.24; 95% CI, 0.23–0.25) and advanced (aHR, 0.47; 95% CI, 0.42–0.54) DKD (Supplementary Table S5).

### 3.8 Sensitivity analyses

We conducted seven sensitivity analyses to confirm the robustness of our findings. In subgroup analyses of patients with overall and advanced DKD, the observed ESRD and all-cause mortality outcomes were predominantly favorable and remained consistent across almost all strata in both Cox and RMST analyses, favoring PRCHMDKD use over nonuse (Supplementary Tables S6, S7). The consistency of the association between PRCHMDKD use and lower risks of ESRD and all-cause mortality remained intact across the various definitions of PRCHMDKD use in patients with overall and advanced DKD (Supplementary Table S8). These associations were withheld when patients with overall and advanced DKD who died or developed ESRD within 30 and 60 days after the index date were excluded (Supplementary Table S9). The positive effects observed in patients with overall and advanced DKD, including ESRD, all-cause mortality, and cardiovascular mortality, remained consistent when variables (such as hypertension, hyperlipidemia, and chronic liver disease) that could potentially influence the utilization of PRCHMDKD between overall DKD/advanced DKD and the PRCHMDKD index date (Supplementary Table S10), two confounding drugs (sodium–glucose cotransporter two inhibitors and glucagon-like peptide-1 agonists) (Supplementary Table S11), and two covariates (stroke and peripheral arterial occlusion disease) (Supplementary Table S12) were incorporate into the regression mode. The beneficial effects of the three outcomes persisted in patients with overall and advanced DKD despite nonusers being defined as those who had never used PRCHMDKD or any other CHMs (Supplementary Table S13).

## 4 Discussion

This is the first study with a substantial sample size and employing robust statistical methods to illustrate the time-dependent cardiorenal and survival benefits associated with the use of sixteen PRCHMDKD, without any accompanying hyperkalemia risk in patients with both overall and advanced DKD. These benefits persisted in diverse sensitivity and subgroup analyses of patients with overall and advanced DKD. Notably, the additive effects of at least four or five classes among the sixteen PRCHMDKD, along with the utilization of multiple items from the sixteen PRCHMDKD, provided enhanced renoprotection for patients with overall and advanced DKD. This suggests the involvement of multiple targets within the oxidative stress pathways in DKD. The most effective renoprotection was observed after the addition of Liu-Wei-Di-Huang-Wan, Liu-Wei-Di-Huang-Wan, *C. chinensis* (Huang-Lian), *R. glutinosa* (Sheng-Di-Huang), *P. lactiflora* (Bai-Shao), *C. officinalis* (Shan-Zhu-Yu), *P. lobata* (Ge-Gen), *A. asphodeloide*s (Zhi-Mu), *L. striatum* (Chuan-Xiong), *A. membranaceus* (Huang-Qi), *S. miltiorrhiza* (Dan-Shan), and *R. palmatum* (Da-Huang), along with Xiao-Chai-Hu-Tang. This composition corresponds to all five classes of the sixteen PRCHMDKD.

Three previous cohort studies based on the NHI data demonstrated renal and survival benefits in incident (Chen et al., 2019; Wu et al., 2021) and advanced (Guo et al., 2021) DKD populations, with only two (Chen et al., 2019; Wu et al., 2021) of these studies showing time-dependent survival benefits. Similar to these studies, our overall findings demonstrated cardiorenal and survival benefits in patients with overall and advanced DKD. In contrast to these studies, our results also help bridge certain knowledge gaps. This study gathered evidence from the literature on effective CHMs for treating DKD, specifically sixteen PRCHMDKD (corresponding to five classes: clearing heat, nourishing yin, dispelling dampness, and tonifying qi), to explore whether the use of these herbal remedies could confer renal and survival benefits and to examine potential time-response and additive effects at both individual and class levels. A meta-analysis on the use of Qiming granule (Liu et al, 2021) and a previous randomized controlled trial on Qidan Dihuang grain (Xiang et al, 2016) revealed significant improvements in renal outcomes. These positive effects were attributed to their properties encompassing three classes: clearing heat, nourishing yin, and tonifying qi. Another randomized controlled trial (Liu et al, 2015) emphasized the effectiveness of spleen-kidney-care Yiqi Huayu and Jiangzhuo decoction, which possesses properties aligned with four classes (clearing heat, nourishing yin, dampness dispelling, and tonifying qi) and demonstrated improvements in clinical syndromes, including reduced proteinuria and blood glucose levels. However, because of the relatively brief follow-up periods and the restricted number of clinical events related to mortality and progression to ESKD, the long-term benefits of these remedies remain uncertain. This study had a longer follow-up period and included more classes of PRCHMDKD, compensating for the shortcomings of the aforementioned studies. Furthermore, the present study is the first to estimate the RMST difference between the PRCHMDKD intervention and control groups, offering a more intuitive measure of the clinical renal and survival benefits associated with PRCHMDKD.

Management of DKD requires a comprehensive approach to address glucose metabolism, oxidative stress, inflammation, and fibrosis. These sixteen PRCHMDKD share common antioxidant, anti-inflammatory, and antifibrotic properties (Tang G. et al., 2021; Chen D. Q. et al., 2022; Lin et al., 2022). The IR/IRS-1/PI3K/GLUT signaling pathway, which is influenced by Huangqi (Astragalus) decoction and the anthraquinone derivative emodin derived from R. palmatum (Da-Huang), plays a pivotal role in regulating blood glucose levels when activated by insulin (Chen et al., 2018; Song et al., 2018). Increased levels of reactive oxygen species (ROS) and inflammation are key factors in renal dysfunction. Ethanol extracts of *R. glutinosa* (Di-Huang) targets the endothelin-1/ROS pathway, reducing oxidative stress and inflammation in the kidney (Liu et al., 2008). Persistent hyperglycemia and AGE-RAGE interactions contribute to intracellular ROS production, which is potentially mitigated by Wu-Ling-San (Liu et al., 2009). Berberine, an isoquinoline alkaloid isolated from *C. chinensis* (Huang-Lian), protects the kidneys by targeting the AGE/RAGE/PKC-β/TGF-β1 pathway (Qiu et al., 2017). Natural isoflavone puerarin derived from *P. lobata* (Ge-Gen) exhibits cardioprotective, antioxidant, and anti-inflammatory effects. It promotes insulin secretion and maintains metabolic balance in STZ-induced diabetic mice, offering anti-diabetic benefits. Moreover, it mitigates diabetes and related complications, such as diabetic nephropathy, by reducing AGE formation and delaying oxidative stress (Jin et al., 2023). *Plantago asiatica* (Che-Qian-Cao) extract possesses anti-inflammatory and antioxidant properties. It safeguards endothelial cells from AGE-induced inflammation and restores cellular function while combating renal oxidative stress (Hong et al., 2011). The water-soluble metabolite Danshensu from *S. miltiorrhiza* (Dan-Shan) and ethanol extract from *L. striatum* (Chuan-Xiong) activate Nrf2, which is crucial for antioxidant defense and oxidative stress mitigation (Tang G. et al., 2021). Renal fibrosis exacerbates DKD. Total glycosides isolated from *R. glutinosa* (Di-Huang), Tanshinone IIA extract from *S. miltiorrhiza* (Dan-Shan), Puerarin identified from *P. lobata* (Ge-Gen), and Ginsenoside Rg1 purified from *P. notoginseng* (Shan-Qi) exhibit anti-fibrotic effects via TGF-β1/Smad2 pathway inhibition (Du et al., 2018; Lin et al., 2022). Xiao-Chai-Hu-Tang prevents TGF-β1 downstream activation (Lin et al., 2012). In DKD, classical fibrotic pathways involving TGF-β and AMPK are related to mitochondrial dysfunction and renal fibrosis (Jha et al., 2018). Catalpol extracted from *R. glutinosa* (Di-Huang) activates the AMPK/SIRT1/NF-κB pathway, and *A. asphodeloides* (Zhi-Mu) increases AMPK phosphorylation, thereby alleviating fibrosis (Lin et al., 2012; Han et al., 2015). Altogether, these mechanisms underscore the multi-targeted approach for renoprotection.

Our findings also indicate a notable reduction in the risk of cardiovascular death among patients with overall and advanced DKD using the sixteen PRCHMDKD. This is consistent with the results of previous cohort studies demonstrating that users of CHM for diabetes experienced a reduced risk of stroke (Lee et al., 2016; Chen D. Q. et al., 2022). Vascular oxidative stress, which is associated with an enhanced inflammatory burden, also contributes to the development and progression of diabetes-related cardiovascular disease and atherosclerosis (Lee et al., 2016; Chen D. Q. et al., 2022). The antioxidant and anti-inflammatory effects of sixteen PRCHMDKD may account for the cardiovascular benefit. Furthermore, the vasodilatory and hypotensive properties of CHMs, such as *S. miltiorrhiza* (Dan-Shan), *A. membranaceus* (Huang-Qi), *R. palmatum* (Da-Huang), and *L. striatum* (Chuan-Xiong), have also been highlighted (Tang F. et al., 2021).

We observed a shorter exposure time of PRCHMDKD in the current study compared to the 17-year study duration. In Taiwan, early management of DKD primarily emphasized Western medicine due to concerns about potential kidney damage from CHM. However, since 2015, a growing body of literature has supported the renoprotective effects of using NHI-covered prescribed CHM in patients with CKD (Lin et al., 2015; Chen et al., 2022b; Chen et al., 2023). Even in 2019, Taiwan’s Ministry of Health and Welfare introduced a traditional Chinese medicine-enhanced CKD outpatient care program (Chen et al., 2023). Consequently, the utilization of prescribed CHM in CKD patients has gradually increased. This may account for the disparity in exposure time between PRCHMDKD and the duration of this study.

In line with prior research illustrating improved survival among patients with both overall (Chen et al., 2019; Wu et al., 2021) and advanced (Guo et al., 2021) DKD who used CHM, our findings indicated decreased risks of cardiovascular and overall mortality in patients with overall and advanced DKD who used PRCHMDKD. Consistent with a prior study (Wu et al., 2021) demonstrating enhanced survival with the use of heat-clearing, yin-nourishing, dampness-dispelling, and qi-tonifying formulas in overall DKD patients, our study observed favorable survival outcomes across all five classes (heat-clearing, yin-nourishing, dampness-dispelling, qi-tonifying, and harmonizing formulas) in patients with both overall and advanced DKD. Notably, the harmonizing formula Xiao-Chai-Hu-Tang exhibited the most favorable outcome, aligning with previous research indicating improved survival among patients with hepatocellular carcinoma (Dai et al., 2016) and cirrhosis (Xie et al., 2020). We et al. (Wu et al., 2021) proposed that the molecular pathways of cell cycle and gene regulation exhibit pronounced differences in coverage between CHM and Western medicine, particularly in G2/M checkpoint-related pathways. CHM may potentially regulate the cell cycle, targeting specific sites for cellular repair, and thereby obtaining potential survival benefits. Given CHM’s multiple targets, its concurrent use with Western medicine offers a complementary treatment approach for DKD.

The study has several significant strengths. First, it used a nationally representative sample to ensure robust statistical power and reliability. Second, the meticulous and comprehensive tracking of study events and prescriptions minimized information and recall bias. Third, various statistical methods were employed, such as propensity score matching to mitigate confounding by indication, competing risk analysis to avoid the overestimation of ESRD, RMST analysis for intuitive estimates, and sensitivity analyses to enhance the reliability and validity of the results. Fourth, this study offered a detailed examination of the time-response and additive effects of sixteen PRCHMDKD and their respective classes concerning cardiorenal and survival outcomes. Fifth, the study addressed the potential risk of hyperkalemia associated with PRCHMDKD. Finally, all scientific Chinese medicines reimbursed by Taiwan’s NHI are overseen by Taiwan’s Committee of Chinese Medicine and Pharmacy of the Ministry of Health and Welfare. They must conform to “Good Manufacturing Practices” standards (Hsieh et al., 2014) and be prescribed by certified CHM practitioners to assure quality and safeguard their public use. This stringent oversight makes the possibility of adulteration and heavy metal contamination of scientific Chinese medicines negligible (Hsieh et al., 2014).

This study has some limitations. First, the study did not assess compliance with prescribed PRCHMDKD or consider pulse and syndromic diagnoses in administrative claims. Although most adverse drug reactions reported from CHMs were mild and related to gastrointestinal system disorders (Chang et al., 2021), adverse drug reaction data (such as herb-herb interactions and herb-drug interactions with Western medicine) were not accessible in the present study. Second, information regarding the use of non-prescribed PRCHMDKD, which refers to obtaining these CHMs without a prescription from a licensed CHM practitioner, usually from traditional pharmacies, was not available in the administrative claim; however, the lower hazard ratio for ESRD associated with prescribed PRCHMDKD supports these findings. Third, caution is needed when applying these results to Western countries, because the PRCHMDKD formulas were provided by Taiwan’s Committee of Chinese Medicine and Pharmacy. Fourth, owing to the complexity of the metabolites, a dose-response relationship could not be evaluated. Fifth, the lack of information on family history, lifestyle factors, weight, blood pressure, sugar levels, and various laboratory data in the NHI claims database limits the assessment of ESRD and death risks. Sixth, being observational, causality could not be established, and unmeasured confounding variables may have affected the results. This is not a substitute for randomized trials. Seventh, in our study, we refrained from breaking down the formulations of Shen-Qi-Wan, Wu-Ling-San, Liu-Wei-Di-Huang-Wan, and Xiao-Chai-Hu-Tang into individual botanical drugs for analysis. Instead, we embraced the holistic utilization principles of traditional Chinese medicine, placing emphasis on the anticipated interactive synergy among metabolites. Eighth, in this study, our focus was on these 16 PRCHMDKD documented in the treatment of DKD. We did not analyze or consider other CHMs outside of these chosen 16, even though some of these alternatives may exhibit less antioxidative action but at higher doses. Additionally, we did not analyze the relationship between the dose of these chosen 16 and the study outcomes. The complexity arises from the fact that these 16 PRCHMDKD reimbursed in Taiwan contain metabolite formulas and are manufactured in powder or tablet forms, making it challenging to accurately assess the precise dose of each PRCHMDKD. Finally, it is important to note that our study, being observational in nature, did not establish causality and may have been influenced by unmeasured confounding variables. Therefore, it should not be considered a substitute for randomized trials.

## 5 Conclusion

Our real-world findings further enhance the existing evidence by underscoring the cardiorenal and survival benefits of using sixteen PRCHMDKD in patients with overall and advanced DKD without an increased risk of hyperkalemia. Moreover, the use of multiple items and categories of PRCHMDKD significantly improved renoprotection in patients with DKD, indicating the involvement of multiple targets within the pathways associated with oxidative stress and inflammation.

## Data Availability

The original contributions presented in the study are included in the article/Supplementary Material, further inquiries can be directed to the corresponding author.

## Abbreviations

DKD, diabetic kidney disease; ESRD, end-stage renal disease; CKD, chronic kidney disease; CHM, Chinese herbal medicines; NHI, National Health Insurance; PRCHMDKD, potentially renoprotective CHM for diabetic kidney disease; LGTD2005; Longitudinal Generation Tracking Database; RMST, restricted mean survival time.
