# Supplementary material for: Sixteen prescribed Chinese herbal medicines provide time-dependent cardiorenal and survival benefits in patients with overall and advanced diabetic kidney disease: a real-world study in Taiwan
Source: Front Pharmacol. 2024 Aug 22;15:1297854. doi: 10.3389/fphar.2024.1297854 (PMC11374620; doi:10.3389/fphar.2024.1297854)

eMethod: To evaluate the therapeutic potential of Chinese herbal medicines (CHMs) in treating diabetic nephropathy, our initial approach involved a systematic and methodical review spanning a diverse range of medical and scientific repositories. This comprehensive investigation included a thorough exploration of pivotal databases such as PubMed, MEDLINE, Embase, and the Cochrane Central Register of Controlled Trials, encompassing records up to July 20, 2023 (refer to search strategies in the following). The subsequent phase of data analysis and synthesis incorporated a detailed examination of academic publications, including systematic meta-analyses, review articles, and randomized controlled trials. There was a specific emphasis on observational studies sourced from Taiwan's National Health Insurance database, with a focus on identifying both monotherapies and composite CHM formulations that underwent human trials.

Search strategies (results of the primary search conducted on July 20, 2023):

PubMed/ MEDLINE

| **Research** | **Command** | **Strategies and keywords** | **Results** |
| --- | --- | --- | --- |
| #1 |  | **Search: Traditional Chinese medicine "medicine, chinese traditional"[MeSH Terms] OR ("medicine"[All Fields] AND "chinese"[All Fields] AND "traditional"[All Fields]) OR "chinese traditional medicine"[All Fields] OR ("traditional"[All Fields] AND "chinese"[All Fields] AND "medicine"[All Fields]) OR "traditional chinese medicine"[All Fields]** | 142,216 |
| #2 |  | **Search: Chinese herbal medicine ("chineses"[All Fields] OR "east asian people"[MeSH Terms] OR ("east"[All Fields] AND "asian"[All Fields] AND "people"[All Fields]) OR "east asian people"[All Fields] OR "chinese"[All Fields]) AND ("plant extracts"[MeSH Terms] OR ("plant"[All Fields] AND "extracts"[All Fields]) OR "plant extracts"[All Fields] OR ("herbal"[All Fields] AND "medicine"[All Fields]) OR "herbal medicine"[All Fields] OR "herbal medicine"[MeSH Terms])** | 77,977 |
| #3 |  | **Search: Herbal medicine "plant extracts"[MeSH Terms] OR ("plant"[All Fields] AND "extracts"[All Fields]) OR "plant extracts"[All Fields] OR ("herbal"[All Fields] AND "medicine"[All Fields]) OR "herbal medicine"[All Fields] OR "herbal medicine"[MeSH Terms] virus 2"[Title/Abstract])) OR ("severe acute respiratory syndrome coronavirus 2"[Title/Abstract]))** | 261,885 |
| #4 |  | **Search: herbal "herbal medicine"[MeSH Terms] OR ("herbal"[All Fields] AND "medicine"[All Fields]) OR "herbal medicine"[All Fields] OR "herbalism"[All Fields] OR "herbal"[All Fields] OR "herbals"[All Fields]** | 102,770 |
| #5 |  | **Search: herb "herb"[All Fields]** | 24,014 |
| #6 |  | **Search:** botanical "botanic"[All Fields] OR "botanical"[All Fields] OR "botanically"[All Fields] OR "botanicals"[All Fields] OR "botanics"[All Fields] | 32,421 |
| #7 | #1 OR #2 OR #3 OR #4 OR #5 OR #6 | **Search: "medicine, chinese traditional"[MeSH Terms] OR ("medicine"[All Fields] AND "chinese"[All Fields] AND "traditional"[All Fields]) OR "chinese traditional medicine"[All Fields] OR ("traditional"[All Fields] AND "chinese"[All Fields] AND "medicine"[All Fields]) OR "traditional chinese medicine"[All Fields] OR (("chineses"[All Fields] OR "east asian people"[MeSH Terms] OR ("east"[All Fields] AND "asian"[All Fields] AND "people"[All Fields]) OR "east asian people"[All Fields] OR "chinese"[All Fields]) AND ("plant extracts"[MeSH Terms] OR ("plant"[All Fields] AND "extracts"[All Fields]) OR "plant extracts"[All Fields] OR ("herbal"[All Fields] AND "medicine"[All Fields]) OR "herbal medicine"[All Fields] OR "herbal medicine"[MeSH Terms])) OR ("plant extracts"[MeSH Terms] OR ("plant"[All Fields] AND "extracts"[All Fields]) OR "plant extracts"[All Fields] OR ("herbal"[All Fields] AND "medicine"[All Fields]) OR "herbal medicine"[All Fields] OR "herbal medicine"[MeSH Terms]) OR ("herbal medicine"[MeSH Terms] OR ("herbal"[All Fields] AND "medicine"[All Fields]) OR "herbal medicine"[All Fields] OR "herbalism"[All Fields] OR "herbal"[All Fields] OR "herbals"[All Fields]) OR "herb"[All Fields] OR ("botanic"[All Fields] OR "botanical"[All Fields] OR "botanically"[All Fields] OR "botanicals"[All Fields] OR "botanics"[All Fields])** | 416,395 |
| #8 |  | **Search:** diabete kidney disease ("diabete"[All Fields] OR "diabetes mellitus"[MeSH Terms] OR ("diabetes"[All Fields] AND "mellitus"[All Fields]) OR "diabetes mellitus"[All Fields] OR "diabetes"[All Fields] OR "diabetes insipidus"[MeSH Terms] OR ("diabetes"[All Fields] AND "insipidus"[All Fields]) OR "diabetes insipidus"[All Fields] OR "diabetic"[All Fields] OR "diabetics"[All Fields] OR "diabets"[All Fields]) AND ("kidney diseases"[MeSH Terms] OR ("kidney"[All Fields] AND "diseases"[All Fields]) OR "kidney diseases"[All Fields] OR ("kidney"[All Fields] AND "disease"[All Fields]) OR "kidney disease"[All Fields]) | 107,647 |
| #9 |  | **Search:** diabete nephropathy ("diabete"[All Fields] OR "diabetes mellitus"[MeSH Terms] OR ("diabetes"[All Fields] AND "mellitus"[All Fields]) OR "diabetes mellitus"[All Fields] OR "diabetes"[All Fields] OR "diabetes insipidus"[MeSH Terms] OR ("diabetes"[All Fields] AND "insipidus"[All Fields]) OR "diabetes insipidus"[All Fields] OR "diabetic"[All Fields] OR "diabetics"[All Fields] OR "diabets"[All Fields]) AND ("kidney diseases"[MeSH Terms] OR ("kidney"[All Fields] AND "diseases"[All Fields]) OR "kidney diseases"[All Fields] OR "nephropathies"[All Fields] OR "nephropathy"[All Fields]) | 104,652 |
| #10 | #8 OR #9 | **Search:** #8 OR #9 (("diabete"[All Fields] OR "diabetes mellitus"[MeSH Terms] OR ("diabetes"[All Fields] AND "mellitus"[All Fields]) OR "diabetes mellitus"[All Fields] OR "diabetes"[All Fields] OR "diabetes insipidus"[MeSH Terms] OR ("diabetes"[All Fields] AND "insipidus"[All Fields]) OR "diabetes insipidus"[All Fields] OR "diabetic"[All Fields] OR "diabetics"[All Fields] OR "diabets"[All Fields]) AND ("kidney diseases"[MeSH Terms] OR ("kidney"[All Fields] AND "diseases"[All Fields]) OR "kidney diseases"[All Fields] OR ("kidney"[All Fields] AND "disease"[All Fields]) OR "kidney disease"[All Fields])) OR (("diabete"[All Fields] OR "diabetes mellitus"[MeSH Terms] OR ("diabetes"[All Fields] AND "mellitus"[All Fields]) OR "diabetes mellitus"[All Fields] OR "diabetes"[All Fields] OR "diabetes insipidus"[MeSH Terms] OR ("diabetes"[All Fields] AND "insipidus"[All Fields]) OR "diabetes insipidus"[All Fields] OR "diabetic"[All Fields] OR "diabetics"[All Fields] OR "diabets"[All Fields]) AND ("kidney diseases"[MeSH Terms] OR ("kidney"[All Fields] AND "diseases"[All Fields]) OR "kidney diseases"[All Fields] OR "nephropathies"[All Fields] OR "nephropathy"[All Fields])) | 116,703 |
| #11 | #7 AND #10 | **Search:** #7 AND #10 ("medicine, chinese traditional"[MeSH Terms] OR ("medicine"[All Fields] AND "chinese"[All Fields] AND "traditional"[All Fields]) OR "chinese traditional medicine"[All Fields] OR ("traditional"[All Fields] AND "chinese"[All Fields] AND "medicine"[All Fields]) OR "traditional chinese medicine"[All Fields]) AND ((("diabete"[All Fields] OR "diabetes mellitus"[MeSH Terms] OR ("diabetes"[All Fields] AND "mellitus"[All Fields]) OR "diabetes mellitus"[All Fields] OR "diabetes"[All Fields] OR "diabetes insipidus"[MeSH Terms] OR ("diabetes"[All Fields] AND "insipidus"[All Fields]) OR "diabetes insipidus"[All Fields] OR "diabetic"[All Fields] OR "diabetics"[All Fields] OR "diabets"[All Fields]) AND ("kidney diseases"[MeSH Terms] OR ("kidney"[All Fields] AND "diseases"[All Fields]) OR "kidney diseases"[All Fields] OR ("kidney"[All Fields] AND "disease"[All Fields]) OR "kidney disease"[All Fields])) OR (("diabete"[All Fields] OR "diabetes mellitus"[MeSH Terms] OR ("diabetes"[All Fields] AND "mellitus"[All Fields]) OR "diabetes mellitus"[All Fields] OR "diabetes"[All Fields] OR "diabetes insipidus"[MeSH Terms] OR ("diabetes"[All Fields] AND "insipidus"[All Fields]) OR "diabetes insipidus"[All Fields] OR "diabetic"[All Fields] OR "diabetics"[All Fields] OR "diabets"[All Fields]) AND ("kidney diseases"[MeSH Terms] OR ("kidney"[All Fields] AND "diseases"[All Fields]) OR "kidney diseases"[All Fields] OR "nephropathies"[All Fields] OR "nephropathy"[All Fields]))) | 1,549 |
| #12 | Review |  | 308 |
| #13 | RCT |  | 71 |
| #14 | Meta-analysis |  | 49 |

EMBASE

| **Research** | **Command** | **Strategies and keywords** | **Results** |
| --- | --- | --- | --- |
| #1 |  | **Search: Traditional Chinese medicine/** | 45,428 |
| #2 |  | **Search: Chinese herbal medicine (Chinese medicine)/** | 57,327 |
| #3 |  | **Search: Herbal medicine/** | 29,591 |
| #4 |  | **Search: herbal (herbal.mp)/** | 88,905 |
| #5 |  | **Search: herb "herb"[All Fields]** | 11,468 |
| #6 |  | **Search:** botanical **(**botanical**.mp)/** | 15,047 |
| #7 | #1 OR #2 OR #3 OR #4 OR #5 OR #6 |  | 172,809 |
| #8 |  | **Search:** diabete kidney disease**/** | 13,162 |
| #9 |  | **Search:** diabete nephropathy**/** | 13,194 |
| #10 | #8 OR #9 |  | 13,194 |
| #11 | #7 AND #10 |  | 0 |

Cochrane Library

| **Research** | **Command** | **Strategies and keywords** | **Results** |
| --- | --- | --- | --- |
| #1 |  | **Search: Traditional Chinese medicine**  Synonyms: Chinese Medicine, Traditional; Chinese Traditional Medicine; Traditional Medicine, Chinese; Traditional Chinese Medicine; Zhong Yi Xue; Chung I Hsueh; Hsueh, Chung I; Traditional Tongue Assessments; Tongue Diagnoses, Traditional; Traditional Tongue Assessment; Tongue Assessment, Traditional; Tongue Diagnosis, Traditional; Traditional Tongue Diagnosis; Traditional Tongue Diagnoses | 10,619 |
| #2 |  | **Search: Chinese herbal medicine**  Synonyms: Chinese Herbal Drugs; Chinese Drugs, Plant; Herbal Drugs, Chinese; Extracts, Chinese Plant; Chinese Plant Extracts; Plant Extracts, Chinese | 3,620 |
| #3 |  | **Search: Herbal medicine**  Synonyms: Herbal Medicine Synonyms: Hawaiian Herbal Medicine; Laau Lapaau; Medicine, Hawaiian Herbal; Herbal Medicine, Hawaiian; La'au Lapa'au; La au Lapa au; Herbalism; Medicine, Herbal Plant Extracts Synonyms: Herbal Medicines; Medicines, Herbal; Plant Extract; Extract, Plant; Extracts, Plant | 6,214 |
| #4 |  | **Search:** **herbal**  Synonyms: Herbals | 11,614 |
| #5 |  | **Search:** **herb**  Phytotherapy Synonyms: Herbal Therapy; Herb Therapy | 2,798 |
| #6 |  | **Search:** botanical  Synonyms: Botanical Antineoplastics; Antineoplastics, Botanical | 507 |
| #7 | #1 OR #2 OR #3 OR #4 OR #5 OR #6 |  | 12,059 |
| #8 |  | **Search:** diabete kidney disease | 8879 |
| #9 |  | **Search:** diabete nephropathy | 5960 |
| #10 | #8 OR #9 |  | 12,600 |
| #11 | #7 AND #10 |  | 19 |
| #12 | Meta-analysis |  | 1 |

eResult: The distribution of CKD stage in propensity-matched overall and advanced DKD patients.

Taiwan’s National Health Insurance (NHI) system uses ICD-9-CM (pre-2016) and ICD-10-CM (post-2016) codes to define diseases, including CKD. Prior to 2016, the exact CKD stage could not be determined using ICD-9-CM codes in the Taiwan NHI dataset. However, patients with anemia and stage 5 CKD could be identified through the use of erythropoiesis-stimulating agents (ESAs), which were reimbursed. Reimbursed ESAs served as a proxy for stage 5 CKD (Chen et al., 2022b), with eligibility criteria including a serum creatinine level exceeding 6 mg/dL and a hematocrit level below 28% before December 2015, and an estimated glomerular filtration rate below 15 ml/min/1.73 m^2^ and hemoglobin below 9 gm/dl after December 2015. The utilization rate of reimbursed ESA reached 85% in 2012. Following 2016, CKD stage classification relied on ICD-10-CM coding. Advanced CKD (stages 4+5) was identified using a combination of ICD-9-CM codes for CKD along with ESA use, ICD-10-CM codes for CKD stage 5 with or without ESA use, and ICD-10-CM codes for CKD stage 4 (Chen et al., 2022b). CKD patients not meeting criteria for advanced CKD were classified as stages 1-3 (mild-to-moderate) based on their ICD-9/10-CM codes for CKD. Hence, the CKD stage (Table 1) in the present study could be approximated as follows: stages 1-3 (mild-to-moderate) *vs.* stages 4-5 (advanced). For propensity-matched overall DKD patients, PRCHMDKD users comprised 41,212 (94.8%) in mild-to-moderate CKD (stages 1-3) and 2268 (5.2%) in advanced CKD (stages 4-5), while PRCHMDKD nonusers consisted of 41,623 (95.7%) in mild-to-moderate CKD and 1857 (4.3%) in advanced CKD. For propensity-matched advanced DKD patients, PRCHMDKD users included 1074 (75.5%) in stage 4 and 348 (24.5%) in stage 5, whereas PRCHMDKD nonusers had 1036 (72.9%) in stage 4 and 386 (27.1%) in stage 5.

| **Supplemental Table S1**. The five functional classifications of 16 potentially renoprotective Chinese herbal medicines for diabetic kidney disease (PRCHMDKD), which are scientific Chinese medicine^#^ | | |
| --- | --- | --- |
| Five Classifications^*^ | Generic Names | Reference |
| **1. Clearing heat** |  |  |
| Sheng-Di-Huang**^†^** | *Rehmannia glutinosa* (Gaertn.) DC. [Orobanchaceae; Radix Rehmanniae] | Hoffman et al., 2020; 顏正華, 1997; Xie et al., 2014 |
| Zhi-Mu | *Anemarrhena asphodeloides* Bunge [Asparagaceae; Rhizoma Anemarrhenae] | 顏正華, 1997 |
| Dan-Shan | *Salvia miltiorrhiza* Bunge [Lamiaceae; Radix et Rhizoma Salviae Miltiorrhizae] | 顏正華, 1997 |
| Da-Huang | *Rheum palmatum* L. [Polygonaceae; Radix et Rhizoma Rhei] | 顏正華, 1997 |
| Huang-Lian | *Coptis chinensis* Franch. [Ranunculaceae; Rhizoma Coptidis] | 顏正華, 1997 |
|  |  |  |
| **2. Nourishing yin** | |  |
| Bai-Shao | *Paeonia lactiflora* Pall. [Paeoniaceae; Radix Paeoniae Alba] | 顏正華, 1997 |
| Shan-Zhu-Yu | *Cornus officinalis* Sieb. et Zucc. [Cornaceae; Fructus Corni] | 顏正華, 1997 |
| Ge-Gen | *Pueraria lobata* (Willd.) Ohwi [Fabaceae; Radix Puerariae] | Dai et al., 2024 |
| Liu-Wei-Di-Huang-Wan | *Rehmannia glutinosa* (Gaertn.) DC. [Orobanchaceae; Radix Rehmanniae], *Cornus officinalis* Sieb. et Zucc. [Cornaceae; Fructus Corni], *Dioscorea oppositifolia* L. [Dioscoreaceae; Rhizoma Dioscoreae], *Paeonia suffruticosa* Andrews [Paeoniaceae; Cortex Moutan], *Poria cocos* (Schw.) Wolf [Polyporaceae; Poria], *Alisma orientale* (Sam.) Juzep. [Alismataceae; Rhizoma Alismatis] | 王&許, 2001 |
|  | |  |
| **3. Dampness dispelling** | |  |
| Chuan-Xiong | *Ligusticum striatum* DC. [Apiaceae; Rhizoma Chuanxiong] | 顏正華, 1997 |
| Che-Qian-Cao | *Plantago asiatica* L. [Plantaginaceae; Herba Plantaginis] | 顏正華, 1997 |
| Shen-Qi-Wan | *Rehmannia glutinosa* (Gaertn.) DC. [Orobanchaceae; Radix Rehmanniae], *Dioscorea oppositifolia* L. [Dioscoreaceae; Rhizoma Dioscoreae], *Cornus officinalis* Sieb. et Zucc. [Cornaceae; Fructus Corni], *Paeonia suffruticosa* Andrews [Paeoniaceae; Cortex Moutan], *Alisma orientale* (Sam.) Juzep. [Alismataceae; Rhizoma Alismatis], *Poria cocos* (Schw.) Wolf [Polyporaceae; Poria], *Aconitum carmichaeli* Debx. [Ranunculaceae; Radix Aconiti Lateralis Preparata], *Cinnamomum cassia* (L.) J.Presl [Lauraceae; Cortex Cinnamomi] | Zhang et al., 2019; Tran et al., 2022 |
| Wu-Ling-San | *Alisma orientale* (Sam.) Juzep. [Alismataceae; Rhizoma Alismatis], *Poria cocos* (Schw.) Wolf [Polyporaceae; Poria], *Atractylodes macrocephala* Koidz. [Asteraceae; Rhizoma Atractylodis Macrocephalae], *Polyporus umbellatus* (Pers.) Fries [Polyporaceae; Polyporus], *Cinnamomum cassia* (L.) J.Presl [Lauraceae; Ramulus Cinnamomi] | 王&許, 2001 |
|  | |  |
| **4. Tonifying qi** | |  |
| Huang-Qi | Astragalus membranaceus (Fisch.) Bunge [Fabaceae; Radix Astragali] | 顏正華, 1997 |
| San-Qi | *Panax notoginseng* (Burkill) F.H. Chen [Araliaceae; Radix Notoginseng] | Xu et al., 2019 |
|  |  |  |
| **5. Harmonizing Formula** |  |  |
| Xiao-Chai-Hu-Tang | Bupleurum chinense DC. [Apiaceae; Radix Bupleuri], Scutellaria baicalensis Georgi [Lamiaceae; Radix Scutellariae], Panax ginseng C.A. Meyer [Araliaceae; Radix Ginseng], Glycyrrhiza uralensis Fisch. [Fabaceae; Radix Glycyrrhizae], Ziziphus jujuba Mill. [Rhamnaceae; Fructus Jujubae], Pinellia ternata (Thunb.) Makino [Araceae; Rhizoma Pinelliae], Zingiber officinale Roscoe [Zingiberaceae; Rhizoma Zingiberis] | 王&許, 2001 |

^*^The 5 classifications of these 16 PRCHMDKD were derived from the latest pharmacological research papers in CHM on DKD and two authoritative textbooks (顏, 1997; 王&許, 2001) of Chinese herbal pharmacopoeia that are designated textbooks for the Chinese Medicine National Examination in Taiwan. We implemented a more adaptable classification approach, guided by the primary pharmacological actions of these 16 PRCHMDKD.

**^†^**Rehmannia (Di-Huang) is known for its heat-clearing and yin-nourishing properties (Zhang et al., 2008). Raw Rehmannia (Sheng-Di-Huang) exhibits a more prominent heat-clearing effect (Hoffman et al., 2020) and is categorized within the heat-clearing class (顏, 1997; Xie et al., 2014), making it a more frequently utilized treatment for DKD (Yokozawa et al., 2004; Lin et al., 2023). Conversely, steamed Rehmannia (Shu-Di-Huang) is known for its yin-nourishing and blood-enriching effects after processing.

^#^The regulation of scientific Chinese medicine in Taiwan, overseen by the Committee of Chinese Medicine and Pharmacy of the Ministry of Health and Welfare, mandates that species of Chinese medicinal materials listed under the Convention on International Trade in Endangered Species of Wild Fauna and Flora must not be used indiscriminately (Accessed 16 Apr 2024: https://dep.mohw.gov.tw/DOCMAP/cp-3190-18448-108.html).

Reference:

Dai, Y.P., Duan, Y., Lu, Y.T., Ni, X.T., Zhang, Y.K., Li, J., et al. (2024). Nourishing Yin traditional Chinese medicine: potential role in the prevention and treatment of type 2 diabetes. *Am J Transl Res.* 16, 234-254.

Hoffman, R.D., Li, C.Y., He, K., Wu, X., He, B.C., He, T.C., et al. (2020). Chinese Herbal Medicine and Its Regulatory Effects on Tumor Related T Cells. *Front Pharmacol.* 11, 492. doi:10.3389/fphar.2020.00492

Lin, M., Zhang, H., Liu, S., Li, A., and Nan, Z. (2023). Efficacy of "Dihuang pill. prescriptions" combined with conventional treatment for diabetic kidney disease: A network meta-analysis and systematic review. *Medicine (Baltimore).* 102, e35290. doi:10.1097/MD.0000000000035290

Tran, D.N.H., Yeh, H.F., Huang, W.J., Wu, P.W., Liao, Y.J., Hwang, S.J., et al. (2022). Efficacy evaluation of Chinese herbal medicine, VGH-BPH1, for patients with benign prostatic hyperplasia: A randomized, double-blind, placebo-controlled, and crossover study. *J Chin Med Assoc.* 85, 639-646. doi:10.1097/JCMA.0000000000000717

Xie, P., Guo, L., Zhao, Y., Bensky, D., and Stoeger, E. (2014). Searching Clue of the Relationship between the Alteration of Bioactive Ingredients and the Herbal “Property” Transformation from Raw Rehmanniae Radix (Sheng-Di-Huang) to Steam-Heating-Processed Rehmanniae Radix (Shu-Di-Huang) by Chromatographic Fingerprint Analysis. *Chin Med.* 5, 47-60. doi: 10.4236/cm.2014.52006

Xu, C., Wang, W., Wang, B., Zhang, T., Cui, X., Pu, Y., et al. (2019). Analytical methods and biological activities of Panax notoginseng saponins: Recent trends. *J Ethnopharmacol.* 236, 443-465. doi:10.1016/j.jep.2019.02.035

Yokozawa, T., Kim, H.Y., and Yamabe, N. (2004). Amelioration of diabetic. nephropathy by dried Rehmanniae Radix (Di Huang) extract. *Am J Chin Med.* 32, 829-839. doi:10.1142/S0192415X04002442

Zhang, J.Y., Hong, C.L., Chen, H.S., Zhou, X.J., Zhang, Y.J., Efferth, T., et al. (2019). Target Identification of Active Constituents of Shen Qi Wan to Treat Kidney Yang Deficiency Using Computational Target Fishing and Network Pharmacology. *Front Pharmacol.* 10, 650. doi:10.3389/fphar.2019.00650

Zhang, R.X., Li, M.X., and Jia, Z.P. (2008). Rehmannia glutinosa: review of botany, chemistry and pharmacology. *J Ethnopharmacol.* 117, 199-214. doi:10.1016/j.jep.2008.02.018

顏正華. (1997). 中藥學. 知音出版社, 台北, ISBN: 957-9101-17-5.

王綿之 & 許濟群. (2001). 方劑學. 知音出版社, 台北, ISBN: 957-9101-66-3.

| **Supplementary Table S2**. The crucial renoprotective mechanisms of these 16 potentially renoprotective Chinese herbal medicines for diabetic kidney disease (PRCHMDKD) | | | |
| --- | --- | --- | --- |
| 16 PRCHMDKD | Renoprotective effects | Mechanisms of metabolites | Reference |
| **1. Clearing heat** |  |  |  |
| Sheng-Di-Huang | glucose/lipid metabolism  antioxidative | Rehmannia glutinosa leaves total glycoside regulates glycolipid level of db/db mice as well as TGF-β/Smad signalling pathway regulation to improve early kidney damage caused by diabetes. | Xu et al., 2020; Waisundara et al., 2008 |
| Zhi-Mu | glucose/lipid metabolism  antioxidative | Anemarrhena asphodeloides Bunge total saponins ameliorate diabetic cardiomyopathy by modifying the PI3K/AKT/HIF-1α pathway to restore glycolytic metabolism. | Chen et al., 2023; Zhong et al., 2024 |
| Dan-Shan | glucose/lipid metabolism  antioxidative  anti-inflammatory  anti-fibrotic activities  podocyte protection | Salvianolic acid A exerts podocyte-protection against MCD injury through PPARγ/Angptl4 and Nrf2/HO-1 pathways and combined with low-dose prednisone possessed a significant anti-proteinuria and therapeutic effects in MCD rats. | Hu et al., 2019; Wang et al., 2019; Xiang et al., 2019 |
| Da-Huang | glucose/lipid metabolism  antioxidative  anti-inflammatory  anti-fibrotic activities | Natural components in rhubarb plays a leading role in the pathogenesis of renal fibrosis, such as the mechanism of the TGF-β/Smad and Wnt/β-catenin signaling pathways. | Zeng et al., 2021; Wang et al., 2022 |
| Huang-Lian | glucose/lipid metabolism  antioxidative  anti-inflammatory  anti-fibrotic activities | Berberine improves inflammatory indicators, such as IL-6 and TNF-α, and oxidative stress indicators, such as the superoxide dismutase activity and malondialdehyde content. | Cui et al., 2018; Hu et al., 2022 |
|  | |  |  |
| **2. Nourishing yin** | |  |  |
| Bai-Shao | antioxidative  anti-inflammatory | Total glucosides of paeony treatment could reduce the albuminuria and inflammatory markers in type 2 diabetes mellitus patients with DKD. | Zhu et al., 2016 |
| Shan-Zhu-Yu | glucose/lipid metabolism  antioxidative  anti-inflammatory  anti-fibrotic activities | Cornus officinalis decreases the levels of 24 h urine protein, blood urea nitrogen (BUN), and Scr in db/db mice by down-regulating the activities of AGEs/RAGE/SphK1 pathway and TGF-β. | Gao et al., 2021; Wu et al., 2023 |
| Ge-Gen | glucose/lipid metabolism  antioxidative  anti-inflammatory | The polysaccharide from Pueraria lobata root exhibits potent antioxidant and anti-inflammatory properties by targeting Nrf2, SIRT1, HMGB1, NF-κB, and NLRP3 of polyphenols, including quercetin, resveratrol, curcumin, and phenolic acid. | Luo et al., 2021; Jin et al., 2023 |
| Liu-Wei-Di-Huang-Wan | antioxidative  anti-inflammatory  anti-fibrotic | The mechanism of action of Liu-Wei-Di-Huang-Wan on DN is mostly related to the TNF signaling pathway as a core mechanism, involving amelioration of angiogenesis, fibrosis, inflammation, disease susceptibility, and oxidative stress. The putative targets identified could be validated through clinical trials. | Chan et al., 2022 |
|  | |  |  |
| **3. Dampness dispelling** | |  |  |
| Chuan-Xiong | antioxidative  anti-inflammatory | The ethanol extract from Ligusticum chuanxiong rhizome acts as an inhibitor of oxidative stress and inflammation through the Nrf2 and NF-κB pathways. | Yang et al., 2018 |
| Che-Qian-Cao | glucose/lipid metabolism  antioxidative | Plantago asiatica L. seed extract effectively improves lipid and glucose metabolism in HF diet-induced obese mice. These effects might be attributed to the upregulation of PPAR signaling. | Yang et al., 2017 |
| Shen-Qi-Wan | glucose/lipid metabolism  antioxidative  anti-fibrotic | Jin-Gui Shen-Qi Wan alleviates renal fibrosis in diabetic nephropathy by downregulating immune complex MHC class II molecules and attenuating the antigen presentation effect of MHC class II on CD4. | Hu et al., 2021; Liang et al., 2024 |
| Wu-Ling-San | antioxidative | Wu-Ling-San powder with conventional symptomatic supportive treatment for DN is effective in alleviating the clinical symptoms, improving renal function, stabilizing FBG, and lowering TG. | Yang et al., 2022 |
|  | |  |  |
| **4. Tonifying qi** | |  |  |
| Huang-Qi | glucose/lipid metabolism  antioxidative  anti-inflammatory  anti-fibrotic  podocyte protection | Astragaloside IV inhibits excessive mesangial cell proliferation and renal fibrosis caused by diabetic nephropathy via modulation of the TGF-β1/Smad/miR-192 signaling pathway. | Li et al., 2011; Mao et al., 2019; Wang et al., 2015 |
| San-Qi | glucose/lipid metabolism  antioxidative  anti-inflammatory  podocyte protection | Ginsenoside Rb1 protects the kidneys from inflammation injuries in DN by upregulating autophagy via suppressing mTOR and activating PINK1/Parkin signaling. | Tang et al., 2020; Wen et al., 2020; He et al., 2022 |
|  | |  |  |
| **5. Harmonizing Formula** | |  |  |
| Xiao-Chai-Hu-Tang | glucose/lipid metabolism  antioxidative | Xiao-Chai-Hu-Tang potentially mediates through decreasing oxidative stress and production of TGF-β1, fibronectin, and collagen IV in the kidney during development of diabetic nephropathy. | Lin et al., 2012 |

Reference:

| Chan, K.W., Yu, K.Y., Yiu, W.H., Xue, R., Lok, S.W., Li, H., et al. (2022). Potential Therapeutic Targets of Rehmannia Formulations on Diabetic Nephropathy: A Comparative Network Pharmacology Analysis. *Front Pharmacol.* 13, 794139. doi:10.3389/fphar.2022.794139  Chen, J., Li, L., Zhang, X., Zhang, Y., Zheng, Q., Lan, M., et al. (2023). Structural characteristics and antioxidant and hypoglycemic activities of a heteropolysaccharide from Anemarrhena asphodeloides Bunge. *Int J Biol Macromol.* 236, 123843. doi:10.1016/j.ijbiomac.2023.123843  Cui, X., Qian, D.W., Jiang, S., Shang, E.X., Zhu, Z.H., and Duan, J.A. (2018). Scutellariae Radix and Coptidis Rhizoma Improve Glucose and Lipid Metabolism in T2DM Rats via Regulation of the Metabolic Profiling and MAPK/PI3K/Akt Signaling Pathway. *Int J Mol Sci.* 19, 3634. doi:10.3390/ijms19113634  Gao, X., Liu, Y., An, Z., and Ni, J. (2021). Active Components and Pharmacological Effects of Cornus officinalis: Literature Review. *Front Pharmacol.* 12, 633447. doi:10.3389/fphar.2021.633447  He, J.Y., Hong, Q., Chen, B.X., Cui, S.Y., Liu, R., Cai, G.Y., et al. (2022). Ginsenoside Rb1 alleviates diabetic kidney podocyte injury by inhibiting aldose reductase activity. *Acta Pharmacol Sin.* 43, 342-353. doi:10.1038/s41401-021-00788-0  Hu, H.C., Zheng, L.T., Yin, H.Y., Tao, Y., Luo, X.Q., Wei, K.S., et al. (2019). A Significant Association Between Rhein and Diabetic Nephropathy in Animals: A Systematic Review and Meta-Analysis. *Front Pharmacol.* 10, 1473. doi:10.3389/fphar.2019.01473  Hu, S., Wang, J., Liu, E., Zhang, X., Xiang, J., Li, W., et al. (2022). Protective effect of berberine in diabetic nephropathy: A systematic review and meta-analysis revealing the mechanism of action. *Pharmacol Res.* 185, 106481. doi:10.1016/j.phrs.2022.106481  Hu, Z., Liu, X., and Yang, M. (2021). Evidence and Potential Mechanisms of Jin-Gui Shen-Qi Wan as a Treatment for Type 2 Diabetes Mellitus: A Systematic Review and Meta-Analysis. *Front Pharmacol.* 12, 699932. doi:10.3389/fphar.2021.699932  Jin, Q., Liu, T., Qiao, Y., Liu, D., Yang, L., Mao, H., et al. (2023). Oxidative stress and inflammation in diabetic nephropathy: role of polyphenols. *Front Immunol.* 14, 1185317. doi:10.3389/fimmu.2023.1185317  Li, M., Wang, W., Xue, J., Gu, Y., and Lin, S. (2011). Meta-analysis of the clinical value of Astragalus membranaceus in diabetic nephropathy. *J Ethnopharmacol.* 133, 412-419. doi:10.1016/j.jep.2010.10.012  Liang, D., Liu, L., Qi, Y., Nan, F., Huang, J., Tang, S., et al. (2024). Jin-Gui-Shen-Qi Wan alleviates fibrosis in mouse diabetic nephropathy via MHC class II. *J Ethnopharmacol.* 324,117745. doi:10.1016/j.jep.2024.117745  Lin, C.C., Lin, L.T., Yen, M.H., Cheng, J.T., Hsing, C.H., and Yeh, C.H. (2012). Renal protective effect of xiao-chai-hu-tang on diabetic nephropathy of type 1-diabetic mice. *Evid Based Complement Alternat Med.* 2012, 984024. doi:10.1155/2012/984024  Luo, D., Dong, X., Huang, J., Huang, C., Fang, G., and Huang, Y. (2021). Pueraria lobata root polysaccharide alleviates glucose and lipid metabolic dysfunction in diabetic db/db mice. *Pharm Biol.* 59, 382-390. doi:10.1080/13880209.2021.1898648  Mao, Q., Chen, C., Liang, H., Zhong, S., Cheng, X., and Li, L. (2019). Astragaloside IV inhibits excessive mesangial cell proliferation and renal fibrosis caused by diabetic nephropathy via modulation of the TGF-beta1/Smad/miR-192 signaling pathway. *Exp Ther Med.* 18, 3053-3061. doi:10.3892/etm.2019.7887  Tang, X., Huang, M., Jiang, J., Liang, X., Li, X., Meng, R., et al. (2020). Panax notoginseng preparations as adjuvant therapy for diabetic kidney disease: a systematic review and meta-analysis. *Pharm Biol.* 58, 138-145. doi:10.1080/13880209.2020.1711782  Waisundara, V.Y., Huang, M., Hsu, A., Huang, D., and Tan, B.K. (2008). Characterization of the anti-diabetic and antioxidant effects of rehmannia glutinosa in streptozotocin-induced diabetic Wistar rats. *Am J Chin Med.* 36, 1083-1104. doi:10.1142/S0192415X08006594  Wang, X., Qi, D., Fu, F., Li, X., Liu, Y., Ji, K., et al. (2019). Therapeutic and antiproteinuric effects of salvianolic acid A in combined with low-dose prednisone in minimal change disease rats: Involvement of PPARgamma/Angptl4 and Nrf2/HO-1 pathways. *Eur J Pharmacol.* 858, 172342. doi:10.1016/j.ejphar.2019.04.023  Wang, Y., Yu, F., Li, A., He, Z., Qu, C., He, C., et al. (2022). The progress and prospect of natural components in rhubarb (Rheum ribes L.) in the treatment of renal fibrosis. *Front Pharmacol.* 13, 919967. doi:10.3389/fphar.2022.919967  Wang, Z.S., Xiong, F., Xie, X.H., Chen, D., Pan, J.H., and Cheng, L. (2015). Astragaloside IV attenuates proteinuria in streptozotocin-induced diabetic nephropathy via the inhibition of endoplasmic reticulum stress. *BMC Nephrol.* 16, 44. doi:10.1186/s12882-015-0031-7  Wen, D., Tan, R.Z., Zhao, C.Y., Li, J.C., Zhong, X., Diao, H., et al. (2020). Astragalus mongholicus Bunge and Panax notoginseng (Burkill) F.H. Chen Formula for Renal Injury in Diabetic Nephropathy-In Vivo and In Vitro Evidence for Autophagy Regulation. *Front Pharmacol.* 11, 732. doi:10.3389/fphar.2020.00732  Wu, C., Wang, J., Zhang, R., Zhao, H., Li, X., Wang, L., et al. (2023). Research progress on Cornus officinalis and its active compounds in the treatment of diabetic nephropathy. *Front Pharmacol.* 14, 1207777. doi:10.3389/fphar.2023.1207777  Xiang, X., Cai, H.D., Su, S.L., Dai, X.X., Zhu, Y., Guo, J.M., et al. (2019). Salvia miltiorrhiza protects against diabetic nephropathy through metabolome regulation and wnt/beta-catenin and TGF-beta signaling inhibition. *Pharmacol Res.* 139, 26-40. doi:10.1016/j.phrs.2018.10.030  Xu, Z., Dai, X.X., Zhang, Q.Y., Su, S.L., Yan, H., Zhu, Y., et al. (2020). Protective effects and mechanisms of Rehmannia glutinosa leaves total glycoside on early kidney injury in db/db mice. *Biomed Pharmacother.* 125, 109926. doi:10.1016/j.biopha.2020.109926  Yang, Q., Qi, M., Tong, R., Wang, D., Ding, L., Li, Z., et al. (2017). Plantago asiatica L. Seed Extract Improves Lipid Accumulation and Hyperglycemia in High-Fat Diet-Induced Obese Mice. *Int J Mol Sci.* 18, 1393. doi:10.3390/ijms18071393  Yang, W.J., Li, Y.R., Gao, H., Wu, X.Y., Wang, X.L., Wang, X.N., et al. (2018). Protective effect of the ethanol extract from Ligusticum chuanxiong rhizome against streptozotocin-induced diabetic nephropathy in mice. *J Ethnopharmacol.* 227, 166-175. doi:10.1016/j.jep.2018.08.037  Yang, Y., Sha, W., Hou, K., Xu, Y., Tan, S., Yin, H., et al. (2022). Efficacy and Safety of Wuling Powder in the Treatment of Patients with Diabetic Nephropathy: A Systematic Review and Meta-Analysis. *Evid Based Complement Alternat Med.*  2022, 1720749. doi:10.1155/2022/1720749  Zeng, J.Y., Wang, Y., Miao, M., and Bao, X.R. (2021). The Effects of Rhubarb for the Treatment of Diabetic Nephropathy in Animals: A Systematic Review and Meta-analysis. *Front Pharmacol.* 12, 602816. doi:10.3389/fphar.2021.602816  Zhong, L., Li, J., Yu, J., Cao, X., Du, J., Liang, L., et al. (2024). Anemarrhena asphodeloides Bunge total saponins ameliorate diabetic cardiomyopathy by modifying the PI3K/AKT/HIF-1alpha pathway to restore glycolytic metabolism. *J Ethnopharmacol.* 319, 117250. doi:10.1016/j.jep.2023.117250  Zhu, Q., Qi, X., Wu, Y., and Wang, K. (2016). Clinical study of total glucosides of paeony for the treatment of diabetic kidney disease in patients with diabetes mellitus. *Int Urol Nephrol.* 48, 1873-1880. doi:10.1007/s11255-016-1345-5   | **Supplementary Table S3.** Median follow-up time (year) | | | | | --- | --- | --- | --- | |  | Users | Nonusers | All | | **Overall DKD patients** |  |  |  | | ESRD | 5.14 | 1.87 | 3.25 | | All death | 5.44 | 2.01 | 3.48 | | CV death | 5.44 | 2.01 | 3.48 | | **Advanced DKD patients** |  |  |  | | ESRD | 6.24 | 3.88 | 4.88 | | All death | 7.20 | 4.54 | 5.75 | | CV death | 7.20 | 4.54 | 5.75 | | Abbreviations: the same as Tables 1-3. | | | |  | **Supplementary Table S4.** Subgroup analysis in terms of the 5 classes of 16 PRCHMDKD for survival outcome in patients with overall and advanced DKD | | | | | | --- | --- | --- | --- | --- | | Taking nonusers as the reference |  | Overall mortality | | | |  | Event / Number | RMST difference (year, 95% CI) | aHR (95% CI) | | Overall DKD patients | | | | | | Clearing heat |  | 8402 / 36578 | 3.00 (2.89, 3.11) | 0.45 (0.43, 0.46) | | Nourishing yin |  | 7938 / 34990 | 3.09 (2.98, 3.21) | 0.44 (0.42, 0.45) | | Dampness dispelling |  | 7363 / 32777 | 3.22 (3.11, 3.34) | 0.43 (0.41, 0.44) | | Tonifying qi |  | 4932 / 21865 | 3.78 (3.64, 3.92) | 0.41 (0.39, 0.42) | | Harmonizing Formula |  | 1093 / 5750 | 6.36 (6.05, 6.68) | 0.34 (0.32, 0.36) | | Advanced DKD patients | | | | | | Clearing heat |  | 204 / 1194 | 1.75 (1.26, 2.24) | 0.54 (0.45, 0.65) | | Nourishing yin |  | 203 / 1179 | 1.67 (1.18, 2.17) | 0.55 (0.46, 0.66) | | Dampness dispelling |  | 194 / 1087 | 1.69 (1.18, 2.20) | 0.56 (0.47, 0.68) | | Tonifying qi |  | 118 / 739 | 2.15 (1.56, 2.73) | 0.48 (0.39, 0.60) | | Harmonizing Formula |  | 27 / 199 | 3.50 (2.44, 4.57) | 0.35 (0.23, 0.52) | | Abbreviations: the same as Tables 1-3.  Adjusted for all covariates (age per year, sex, comorbidities, number of medical visits, Charlson comorbidity index, and confounding drugs) and competing risk for ESRD. | | | | | | | | |
| --- | --- | --- | --- | --- | --- | --- | --- | --- | --- | --- | --- | --- | --- | --- | --- | --- | --- | --- | --- | --- | --- | --- | --- | --- | --- | --- | --- | --- | --- | --- | --- | --- | --- | --- | --- | --- | --- | --- | --- | --- | --- | --- | --- | --- | --- | --- | --- | --- | --- | --- | --- | --- | --- | --- | --- | --- | --- | --- | --- | --- | --- | --- | --- | --- | --- | --- | --- | --- | --- | --- | --- | --- | --- | --- | --- | --- | --- | --- | --- | --- | --- | --- | --- | --- | --- | --- | --- | --- | --- | --- | --- | --- | --- | --- | --- | --- | --- | --- | --- | --- | --- | --- | --- | --- | --- | --- | --- | --- | --- | --- | --- | --- | --- | --- | --- | --- | --- | --- | --- | --- | --- | --- | --- | --- | --- | --- |
| **Supplementary Table S5.** Hyperkalemia occurrence during follow-up | | | |
|  |  | Users | Nonusers |
| **Overall DKD patients** |  | n=43480 | n=43480 |
| Inpatient and outpatient events |  | 4211 / 2382 | 3287 / 1838 |
| Person-years observed |  | 27886.5 | 14532.5 |
| Incidence rate per 100 person-years |  | 0.15 | 0.23 |
| Adjusted incidence rate ratio^*^ (95% CI) |  | 0.24 (0.23, 0.25) | 1 (reference) |
| **Advanced DKD patients** |  | n=1422 | n=1422 |
| Inpatient and outpatient events |  | 521 / 225 | 498 / 224 |
| Person-years observed |  | 1090.93 | 803.8 |
| Incidence rate per 100 person-years |  | 0.48 | 0.62 |
| Adjusted incidence rate ratio^*^ (95% CI) |  | 0.47 (0.42, 0.54) | 1 (reference) |
| Abbreviations: the same as Tables 1-3.  ^*^Adjusted for all covariates (age per year, sex, comorbidities, number of medical visits, Charlson comorbidity index, confounding drugs). | | | |

| **Supplementary Table S6.** Subgroup analyses in patients with overall DKD | | | | | | | | | | | | | | | |
| --- | --- | --- | --- | --- | --- | --- | --- | --- | --- | --- | --- | --- | --- | --- | --- |
|  | ESRD | | | | | | |  | Overall mortality | | | | | | |
|  | Use (*vs.* Nonuse) | | |  | Use (*vs.* Nonuse) | | |  | Use (*vs.* Nonuse) | | |  | Use (*vs.* Nonuse) | | |
| Variable | aHR | 95% CI | *p*-value |  | RMSTd | 95% CI | *p*-value |  | aHR | 95% CI | *p*-value |  | RMSTd | 95% CI | *p*-value |
| Sex |  |  |  |  |  |  |  |  |  |  |  |  |  |  |  |
| Male | 0.76 | 0.69-0.84 | <0.0001 |  | 0.17 | 0.07-0.27 | 0.0008 |  | 0.52 | 0.50-0.54 | <0.0001 |  | 2.49 | 2.34-2.65 | <0.0001 |
| Female | 0.57 | 0.52-0.63 | <0.0001 |  | 0.44 | 0.34-0.54 | <0.0001 |  | 0.44 | 0.42-0.46 | <0.0001 |  | 2.90 | 2.76-3.05 | <0.0001 |
| Age (year) |  |  |  |  |  |  |  |  |  |  |  |  |  |  |  |
| ≤60 | 0.65 | 0.59-0.71 | <0.0001 |  | 0.38 | 0.28-0.48 | <0.0001 |  | 0.46 | 0.44-0.49 | <0.0001 |  | 1.85 | 1.70-1.99 | <0.0001 |
| >60 | 0.66 | 0.60-0.73 | <0.0001 |  | 0.26 | 0.16-0.35 | <0.0001 |  | 0.49 | 0.47-0.50 | <0.0001 |  | 2.94 | 2.80-3.08 | <0.0001 |
| Hypertension |  |  |  |  |  |  |  |  |  |  |  |  |  |  |  |
| No | 0.67 | 0.58-0.79 | <0.0001 |  | 0.25 | 0.15-0.36 | <0.0001 |  | 0.49 | 0.46-0.52 | <0.0001 |  | 1.99 | 1.81-2.17 | <0.0001 |
| Yes | 0.65 | 0.61-0.71 | <0.0001 |  | 0.32 | 0.23-0.41 | <0.0001 |  | 0.48 | 0.46-0.49 | <0.0001 |  | 2.87 | 2.74-3.00 | <0.0001 |
| Coronary heart disease |  |  |  |  |  |  |  |  |  |  |  |  |  |  |  |
| No | 0.66 | 0.61-0.71 | <0.0001 |  | 0.31 | 0.23-0.39 | <0.0001 |  | 0.47 | 0.46-0.49 | <0.0001 |  | 2.52 | 2.40-2.64 | <0.0001 |
| Yes | 0.65 | 0.56-0.76 | <0.0001 |  | 0.31 | 0.15-0.48 | 0.0002 |  | 0.49 | 0.46-0.51 | <0.0001 |  | 2.96 | 2.73-3.18 | <0.0001 |
| Hyperlipidemia |  |  |  |  |  |  |  |  |  |  |  |  |  |  |  |
| No | 0.70 | 0.64-0.76 | <0.0001 |  | 0.28 | 0.19-0.37 | <0.0001 |  | 0.46 | 0.45-0.48 | <0.0001 |  | 3.01 | 2.88-3.14 | <0.0001 |
| Yes | 0.61 | 0.55-0.68 | <0.0001 |  | 0.35 | 0.23-0.46 | <0.0001 |  | 0.51 | 0.48-0.53 | <0.0001 |  | 2.25 | 2.07-2.42 | <0.0001 |
| Chronic liver  disease |  |  |  |  |  |  |  |  |  |  |  |  |  |  |  |
| No | 0.67 | 0.63-0.73 | <0.0001 |  | 0.31 | 0.22-0.39 | <0.0001 |  | 0.49 | 0.47-0.50 | <0.0001 |  | 2.71 | 2.59-2.83 | <0.0001 |
| Yes | 0.58 | 0.49-0.69 | <0.0001 |  | 0.32 | 0.20-0.45 | <0.0001 |  | 0.45 | 0.43-0.48 | <0.0001 |  | 2.75 | 2.53-2.96 | <0.0001 |
| ACEI/ARB |  |  |  |  |  |  |  |  |  |  |  |  |  |  |  |
| No | 0.67 | 0.60-0.75 | <0.0001 |  | 0.22 | 0.15-0.30 | <0.0001 |  | 0.47 | 0.45-0.49 | <0.0001 |  | 2.59 | 2.46-2.73 | <0.0001 |
| Yes | 0.65 | 0.59-0.71 | <0.0001 |  | 0.40 | 0.27-0.53 | <0.0001 |  | 0.49 | 0.47-0.51 | <0.0001 |  | 2.73 | 2.56-2.90 | <0.0001 |
| NSAID |  |  |  |  |  |  |  |  |  |  |  |  |  |  |  |
| No | 0.67 | 0.59-0.76 | <0.0001 |  | 0.36 | 0.20-0.52 | <0.0001 |  | 0.54 | 0.51-0.57 | <0.0001 |  | 2.30 | 2.08-2.51 | <0.0001 |
| Yes | 0.65 | 0.60-0.70 | <0.0001 |  | 0.29 | 0.21-0.36 | <0.0001 |  | 0.46 | 0.45-0.48 | <0.0001 |  | 2.83 | 2.71-2.96 | <0.0001 |
| Anti-hyperglycemic drugs |  |  |  |  |  |  |  |  |  |  |  |  |  |  |  |
| No | 0.70 | 0.56-0.88 | 0.0024 |  | 0.08 | 0.02-0.13 | 0.009 |  | 0.47 | 0.44-0.49 | <0.0001 |  | 2.35 | 2.18-2.52 | <0.0001 |
| Yes | 0.65 | 0.61-0.70 | <0.0001 |  | 0.45 | 0.34-0.56 | <0.0001 |  | 0.48 | 0.47-0.5 | <0.0001 |  | 2.85 | 2.72-2.99 | <0.0001 |
| CCI |  |  |  |  |  |  |  |  |  |  |  |  |  |  |  |
| <3 | 0.67 | 0.61-0.73 | <0.0001 |  | 0.28 | 0.20-0.36 | <0.0001 |  | 0.50 | 0.48-0.52 | <0.0001 |  | 2.30 | 2.17-2.43 | <0.0001 |
| ≥3 | 0.66 | 0.60-0.74 | <0.0001 |  | 0.36 | 0.23-0.49 | <0.0001 |  | 0.46 | 0.44-0.48 | <0.0001 |  | 3.11 | 2.94-3.28 | <0.0001 |
| Medical visits |  |  |  |  |  |  |  |  |  |  |  |  |  |  |  |
| ≤24 | 0.73 | 0.66-0.79 | <0.0001 |  | 0.24 | 0.15-0.33 | <0.0001 |  | 0.52 | 0.50-0.54 | <0.0001 |  | 2.21 | 2.07-2.35 | <0.0001 |
| >24 | 0.57 | 0.51-0.63 | <0.0001 |  | 0.41 | 0.30-0.52 | <0.0001 |  | 0.44 | 0.43-0.46 | <0.0001 |  | 3.23 | 3.06-3.39 | <0.0001 |
| Abbreviations: the same as Tables 1-3, RMSTd, RMST difference.  Adjusted for all covariates (age per year, sex, comorbidities, number of medical visits, Charlson comorbidity index, NSAID, and ACEI/ARB) and competing mortality for ESRD. | | | | | | | | | | | | | | | |

| **Supplementary Table S7.** Subgroup analyses in patients with advanced DKD | | | | | | | | | | | | | | | |
| --- | --- | --- | --- | --- | --- | --- | --- | --- | --- | --- | --- | --- | --- | --- | --- |
|  | ESRD | | | | | | |  | Overall mortality | | | | | | |
|  | Use (*vs.* Nonuse) | | |  | Use (*vs.* Nonuse) | | |  | Use (*vs.* Nonuse) | | |  | Use (*vs.* Nonuse) | | |
| Variable | aHR | 95% CI | *p*-value |  | RMSTd | 95% CI | *p*-value |  | aHR | 95% CI | *p*-value |  | RMSTd | 95% CI | *p*-value |
| Sex |  |  |  |  |  |  |  |  |  |  |  |  |  |  |  |
| Male | 0.67 | 0.49-0.93 | 0.016 |  | 0.34 | -0.31-0.98 | 0.30 |  | 0.63 | 0.49-0.80 | 0.0001 |  | 1.28 | 0.64-1.93 | 0.0001 |
| Female | 0.83 | 0.61-1.11 | 0.21 |  | 0.98 | 0.25-1.70 | 0.008 |  | 0.56 | 0.44-0.72 | <0.0001 |  | 1.78 | 1.09-2.47 | <0.0001 |
| Age (year) |  |  |  |  |  |  |  |  |  |  |  |  |  |  |  |
| ≤60 | 0.75 | 0.55-1.04 | 0.08 |  | 0.69 | -0.16-1.53 | 0.11 |  | 0.64 | 0.44-0.92 | 0.015 |  | 0.72 | 0.08-1.37 | 0.028 |
| >60 | 0.81 | 0.36-1.80 | 0.60 |  | 0.58 | 0.02-1.14 | 0.043 |  | 0.59 | 0.48-0.72 | <0.0001 |  | 1.79 | 1.15-2.43 | <0.0001 |
| Hypertension |  |  |  |  |  |  |  |  |  |  |  |  |  |  |  |
| No | 0.82 | 0.65-1.05 | 0.11 |  | 1.26 | 0.11-2.41 | 0.032 |  | 0.45 | 0.28-0.74 | 0.002 |  | 1.58 | 0.55-2.61 | 0.003 |
| Yes | 0.75 | 0.59-0.95 | 0.017 |  | 0.50 | -0.03-1.03 | 0.07 |  | 0.60 | 0.50-0.73 | <0.0001 |  | 1.54 | 1.00-2.07 | <0.0001 |
| Coronary heart  disease |  |  |  |  |  |  |  |  |  |  |  |  |  |  |  |
| No | 1.13 | 0.64-2.00 | 0.67 |  | 0.81 | 0.25-1.38 | 0.005 |  | 0.58 | 0.47-0.71 | <0.0001 |  | 1.50 | 0.97-2.02 | <0.0001 |
| Yes | 0.81 | 0.60-1.10 | 0.18 |  | -0.02 | -0.91-0.88 | 0.97 |  | 0.63 | 0.45-0.88 | 0.007 |  | 1.50 | 0.44-2.55 | 0.006 |
| Hyperlipidemia |  |  |  |  |  |  |  |  |  |  |  |  |  |  |  |
| No | 0.80 | 0.59-1.09 | 0.16 |  | 0.68 | 0.02-1.34 | 0.044 |  | 0.53 | 0.42-0.68 | <0.0001 |  | 1.58 | 0.95-2.21 | <0.0001 |
| Yes | 0.91 | 0.72-1.15 | 0.44 |  | 0.59 | -0.11-1.29 | 0.10 |  | 0.63 | 0.49-0.80 | 0.0002 |  | 1.50 | 0.77-2.23 | <0.0001 |
| Chronic liver  disease |  |  |  |  |  |  |  |  |  |  |  |  |  |  |  |
| No | 0.40 | 0.22-0.73 | 0.003 |  | 0.31 | -0.22-0.84 | 0.25 |  | 0.59 | 0.49-0.72 | <0.0001 |  | 1.52 | 1.00-2.04 | <0.0001 |
| Yes | 0.66 | 0.46-0.97 | 0.032 |  | 1.98 | 0.78-3.19 | 0.001 |  | 0.55 | 0.36-0.86 | 0.009 |  | 1.37 | 0.23-2.50 | 0.018 |
| ACEI/ARB |  |  |  |  |  |  |  |  |  |  |  |  |  |  |  |
| No | 0.89 | 0.68-1.16 | 0.38 |  | 0.90 | 0.15-1.65 | 0.018 |  | 0.52 | 0.39-0.71 | <0.0001 |  | 1.49 | 0.78-2.19 | <0.0001 |
| Yes | 0.85 | 0.56-1.30 | 0.45 |  | 0.43 | -0.19-1.05 | 0.18 |  | 0.61 | 0.50-0.76 | <0.0001 |  | 1.53 | 0.90-2.17 | <0.0001 |
| NSAID |  |  |  |  |  |  |  |  |  |  |  |  |  |  |  |
| No | 0.77 | 0.60-1.00 | 0.046 |  | 0.57 | -0.48-1.62 | 0.29 |  | 0.48 | 0.33-0.69 | 0.0001 |  | 1.74 | 0.78-2.70 | 0.0004 |
| Yes | 0.87 | 0.66-1.13 | 0.30 |  | 0.64 | 0.10-1.18 | 0.02 |  | 0.63 | 0.52-0.77 | <0.0001 |  | 1.38 | 0.83-1.93 | <0.0001 |
| Anti-hyperglycemic drugs |  |  |  |  |  |  |  |  |  |  |  |  |  |  |  |
| No | 0.80 | 0.64-1.00 | 0.05 |  | 0.13 | -0.79-1.06 | 0.78 |  | 0.68 | 0.43-1.05 | 0.08 |  | 1.09 | -0.03-2.21 | 0.06 |
| Yes | 0.70 | 0.41-1.18 | 0.18 |  | 0.70 | 0.15-1.25 | 0.012 |  | 0.57 | 0.47-0.69 | <0.0001 |  | 1.58 | 1.05-2.10 | <0.0001 |
| CCI |  |  |  |  |  |  |  |  |  |  |  |  |  |  |  |
| <3 | 0.73 | 0.51-1.04 | 0.08 |  | 0.60 | -0.12-1.32 | 0.10 |  | 0.58 | 0.45-0.75 | <0.0001 |  | 1.42 | 0.79-2.06 | <0.0001 |
| ≥3 | 0.87 | 0.66-1.16 | 0.35 |  | 0.53 | -0.10-1.16 | 0.10 |  | 0.60 | 0.47-0.76 | <0.0001 |  | 1.58 | 0.87-2.28 | <0.0001 |
| Medical visits |  |  |  |  |  |  |  |  |  |  |  |  |  |  |  |
| ≤24 | 0.71 | 0.50-1.01 | 0.06 |  | 0.45 | -0.24-1.14 | 0.20 |  | 0.56 | 0.43-0.73 | <0.0001 |  | 1.36 | 0.74-1.97 | <0.0001 |
| >24 | 0.61 | 0.48-0.77 | <0.0001 |  | 0.81 | 0.15-1.46 | 0.016 |  | 0.61 | 0.48-0.77 | <0.0001 |  | 1.55 | 0.82-2.28 | <0.0001 |
| Abbreviations: the same as Tables 1-3, RMSTd, RMST difference.  Adjusted for all covariates (age per year, sex, comorbidities, number of medical visits, Charlson comorbidity index, NSAID, and ACEI/ARB) and competing mortality for ESRD. | | | | | | | | | | | | | | | |

| **Supplementary Table S8.** Study outcomes by definitions of 16 PRCHMDKD use in patients with overall and advanced DKD | | | | | | | | |
| --- | --- | --- | --- | --- | --- | --- | --- | --- |
|  |  | ESRD | | |  | Overall mortality | | |
|  |  | Event/Number | RMSTd  (y, 95% CI) | aHR  (95% CI) |  | Event/Number | RMSTd  (y, 95% CI) | aHR  (95% CI) |
| **Overall DKD patients** | | | | | | | | |
| Over 30 days as use | |  |  |  |  |  |  |  |
| Nonuse (n=29905) | | 1114 | 0 (Reference) | 1 (Reference) |  | 7814 | 0 (Reference) | 1 (Reference) |
| Use (n=29905) | | 1154 | 0.45 (0.37, 0.53) | 0.52 (0.47, 0.56) |  | 6614 | 3.2 (3.08, 3.32) | 0.39 (0.38, 0.41) |
| Over 60 days as use | |  |  |  |  |  |  |  |
| Nonuse (n=22978) | | 889 | 0 (Reference) | 1 (Reference) |  | 5989 | 0 (Reference) | 1 (Reference) |
| Use (n=22978) | | 828 | 0.56 (0.47, 0.64) | 0.44 (0.40, 0.49) |  | 4855 | 3.43 (3.30, 3.56) | 0.36 (0.34, 0.37) |
| **Advanced DKD patients** | | | | | | | | |
| Over 30 days as use | |  |  |  |  |  |  |  |
| Nonuse (n=1011) | | 121 | 0 (Reference) | 1 (Reference) |  | 204 | 0 (Reference) | 1 (Reference) |
| Use (n=1011) | | 111 | 0.96 (0.42, 1.5) | 0.67 (0.52, 0.86) |  | 166 | 1.84 (1.31, 2.37) | 0.50 (0.41, 0.62) |
| Over 60 days as use | |  |  |  |  |  |  |  |
| Nonuse (n=802) | | 97 | 0 (Reference) | 1 (Reference) |  | 155 | 0 (Reference) | 1 (Reference) |
| Use (n=802) | | 82 | 1.2 (0.62, 1.79) | 0.58 (0.43, 0.78) |  | 133 | 1.7 (1.11, 2.28) | 0.52 (0.41, 0.66) |
| Abbreviation: the same as Tables 2-6.  Adjusted for all covariates (age per year, sex, comorbidities, number of medical visits, Charlson comorbidity index, confounding drugs) and competing risk for ESRD. | | | | | | | | |

| **Supplementary Table S9.** Risks of study outcomes in patients with overall and advanced DKD excluding dying or developing ESRD within 30 and 60 days after the index date | | | | | | | | | | | | |
| --- | --- | --- | --- | --- | --- | --- | --- | --- | --- | --- | --- | --- |
|  |  | ESRD | | | | |  | Overall mortality | | | | |
|  |  | Event/Number | RMSTd  (y, 95% CI) | | | aHR  (95% CI) |  | Event/Number | | RMSTd  (y, 95% CI) | aHR  (95% CI) | |
| **All DKD patients** | | | | | | | | | | | | |
| Follow-up >30 days | |  |  | | |  |  |  | |  |  | |
| Nonuse | | 1507/41305 | 0 (Reference) | | | 1 (Reference) |  | 10712/41410 | | 0 (Reference) | 1 (Reference) | |
| Use | | 1802/42992 | 0.29 (0.22, 0.36) | | | 0.67 (0.62, 0.72) |  | 10138/43046 | | 2.59 (2.49, 2.7) | 0.49 (0.48, 0.51) | |
| Follow-up >60 days | |  |  | | |  |  |  | |  |  | |
| Nonuse | | 1434/39581 | 0 (Reference) | | | 1 (Reference) |  | 10314/39759 | | 0 (Reference) | 1 (Reference) | |
| Use | | 1769/42538 | 0.27 (0.2, 0.34) | | | 0.67 (0.62, 0.72) |  | 10032/42625 | | 2.51 (2.41, 2.62) | 0.50 (0.49, 0.51) | |
| **Advanced DKD patients** | | | | | | | | | | | | |
| Follow-up >30 days | |  |  | | |  |  |  | |  |  | |
| Nonuse | | 163/1401 | 0 (Reference) | | | 1 (Reference) |  | 278/1404 | | 0 (Reference) | 1 (Reference) | |
| Use | | 164/1406 | 0.64 (0.16, 1.13) | | | 0.78 (0.63, 0.97) |  | 247/1412 | | 1.48 (1.01, 1.96) | 0.59 (0.5, 0.71) | |
| Follow-up >60 days | |  |  | | |  |  |  | |  |  | |
| Nonuse | | 161/1372 | 0 (Reference) | | | 1 (Reference) |  | 274/1377 | | 0 (Reference) | 1 (Reference) | |
| Use | | 161/1399 | 0.65 (0.17, 1.13) | | | 0.77 (0.62, 0.96) |  | 247/1408 | | 1.45 (0.98, 1.93) | 0.60 (0.51, 0.72) | |
| Abbreviation: the same as Tables 2-6, RMSTd, RMST difference.  Adjusted for all covariates (age per year, sex, comorbidities, number of medical visits, Charlson comorbidity index, confounding drugs) and competing risk for ESRD. | | | | | | | | | | | | |
| **Supplementary Table S10.** Sensitivity analysis: Incorporating variables (such as hypertension, hyperlipidemia, and chronic liver disease) that could potentially influence the utilization of PRCHMDKD between overall DKD/advanced DKD and the PRCHMDKD index date into the regression model | | | | | | | | | | | |  |
|  | | | |  | RMST difference  (year, 95% CI) | | | | Adjusted HR  (95% CI) | | |  |
| **Overall DKD patients** | | | |  |  | | | |  | | |  |
| ESRD | | | |  | 0.31 (0.24, 0.38) | | | | 0.66 (0.62, 0.71) | | |  |
| All death | | | |  | 2.74 (2.64, 2.85) | | | | 0.48 (0.47, 0.49) | | |  |
| CV death | | | |  | 1.21 (1.12, 1.31) | | | | 0.50 (0.48, 0.53) | | |  |
| **Advanced DKD patients** | | | |  |  | | | |  | | |  |
| ESRD | | | |  | 0.51 (0, 1.02) | | | | 0.81 (0.64, 1.02) | | |  |
| All death | | | |  | 1.34 (0.84, 1.84) | | | | 0.61 (0.51, 0.74) | | |  |
| CV death | | | |  | 0.41 (0.03, 0.80) | | | | 0.69 (0.49, 0.96) | | |  |
| Abbreviations: the same as Tables 1-3.  Adjusted for all covariates (age per year, sex, comorbidities, number of medical visits, Charlson comorbidity index, confounding drugs) and three variables (hypertension, hyperlipidemia, and chronic liver disease) between overall DKD/advanced DKD and the PRCHMDKD index date that could potentially influence the utilization of PRCHMDKD. | | | | | | | | | | | |  |

| **Supplementary Table S11.** Sensitivity analysis: Incorporating two confounding drugs (sodium–glucose cotransporter 2 inhibitors and glucagon-like peptide*-*1 agonists) into the regression model | | | |
| --- | --- | --- | --- |
|  |  | RMST difference  (year, 95% CI) | Adjusted HR  (95% CI) |
| **Overall DKD patients** |  |  |  |
| ESRD |  | 0.31 (0.24, 0.38) | 0.66 (0.61, 0.70) |
| All death |  | 2.71 (2.60, 2.81) | 0.48 (0.47, 0.49) |
| CV death |  | 1.18 (1.09, 1.28) | 0.51 (0.48, 0.53) |
| **Advanced DKD patients** |  |  |  |
| ESRD |  | 0.50 (-0.01, 1.01) | 0.84 (0.67, 1.06) |
| All death |  | 1.37 (0.87, 1.87) | 0.61 (0.51, 0.74) |
| CV death |  | 0.42 (0.04, 0.81) | 0.69 (0.50, 0.96) |
| Abbreviations: the same as Tables 1-3.  Adjusted for all covariates (age per year, sex, comorbidities, number of medical visits, Charlson comorbidity index, confounding drugs) and two confounding drugs (sodium–glucose cotransporter 2 inhibitors and glucagon-like peptide*-*1 agonists). | | | |

| **Supplementary Table S12.** Sensitivity analysis: Incorporating two covariates (stroke and peripheral arterial occlusion disease) into the regression model | | | |
| --- | --- | --- | --- |
|  |  | RMST difference  (year, 95% CI) | Adjusted HR  (95% CI) |
| **Overall DKD patients** |  |  |  |
| ESRD |  | 0.31 (0.24, 0.38) | 0.66 (0.61, 0.70) |
| All death |  | 2.68 (2.57, 2.78) | 0.48 (0.47, 0.49) |
| CV death |  | 1.16 (1.06, 1.25) | 0.50 (0.48, 0.53) |
| **Advanced DKD patients** |  |  |  |
| ESRD |  | 0.48 (-0.03, 1.00) | 0.84 (0.66, 1.05) |
| All death |  | 1.38 (0.88, 1.88) | 0.62 (0.51, 0.75) |
| CV death |  | 0.43 (0.05, 0.81) | 0.69 (0.50, 0.96) |
| Abbreviations: the same as Tables 1-3.  Adjusted for all covariates (age per year, sex, comorbidities, number of medical visits, Charlson comorbidity index, confounding drugs) and two covariates (stroke and peripheral arterial occlusion disease). | | | |

| **Supplementary Table S13.** Sensitivity analysis: Nonusers were defined as patients with DKD/advanced DKD who had never utilized PRCHMDKD or any other CHMs | | | |
| --- | --- | --- | --- |
|  |  | RMST difference  (year, 95% CI) | Adjusted HR  (95% CI) |
| **Overall DKD patients (42573 PRCHMDKD users and 42573 nonusers)^†^** | | | |
| ESRD |  | 0.32 (0.25, 0.39) | 0.65 (0.60, 0.69) |
| All death |  | 2.79 (2.68, 2.90) | 0.47 (0.46, 0.48) |
| CV death |  | 1.21 (1.12, 1.31) | 0.50 (0.47, 0.52) |
| **Advanced DKD patients (1364 PRCHMDKD users and 1364 nonusers)^†^** | | | |
| ESRD |  | 0.52 (0.03, 1.01) | 0.82 (0.66, 1.02) |
| All death |  | 1.81 (1.33, 2.30) | 0.52 (0.44, 0.63) |
| CV death |  | 0.70 (0.34, 1.06) | 0.53 (0.38, 0.73) |
| Abbreviations: the same as Tables 1-3.  Adjusted for all covariates (age per year, sex, comorbidities, number of medical visits, Charlson comorbidity index, confounding drugs) and two covariates (stroke and peripheral arterial occlusion disease).  ^†^All values of standardized mean difference in baseline characteristics (listed in Table 1) between propensity score-matched PRCHMDKD users and nonusers were less than 0.1, indicating that a greater between-group comparability was achieved. | | | |

Supplementary Figure S1. Cumulative incidences of (A) end-stage renal disease (ESRD) and (B) overall mortality among PRCHMDKD users and nonusers in patients with overall DKD.


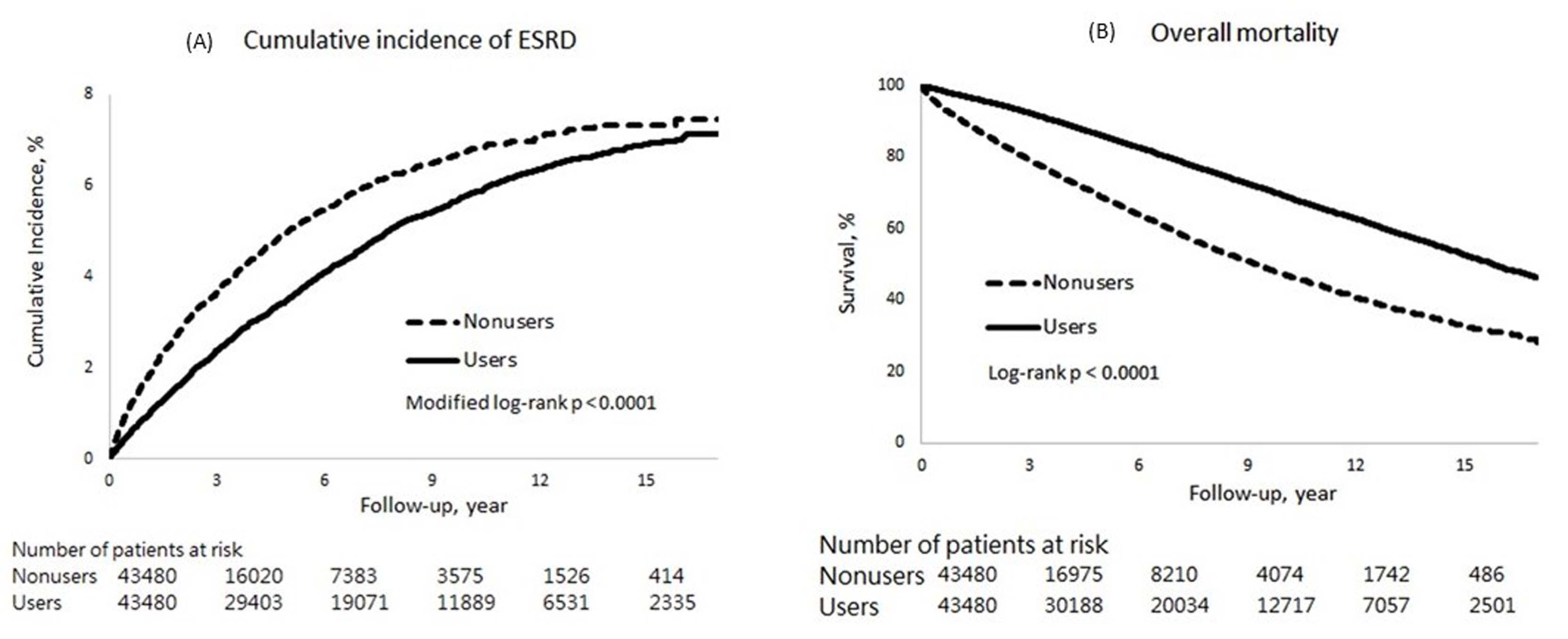


Supplementary Figure S2. Cumulative incidences of (A) end-stage renal disease (ESRD) and (B) overall mortality among PRCHMDKD users and nonusers in patients with advanced DKD.


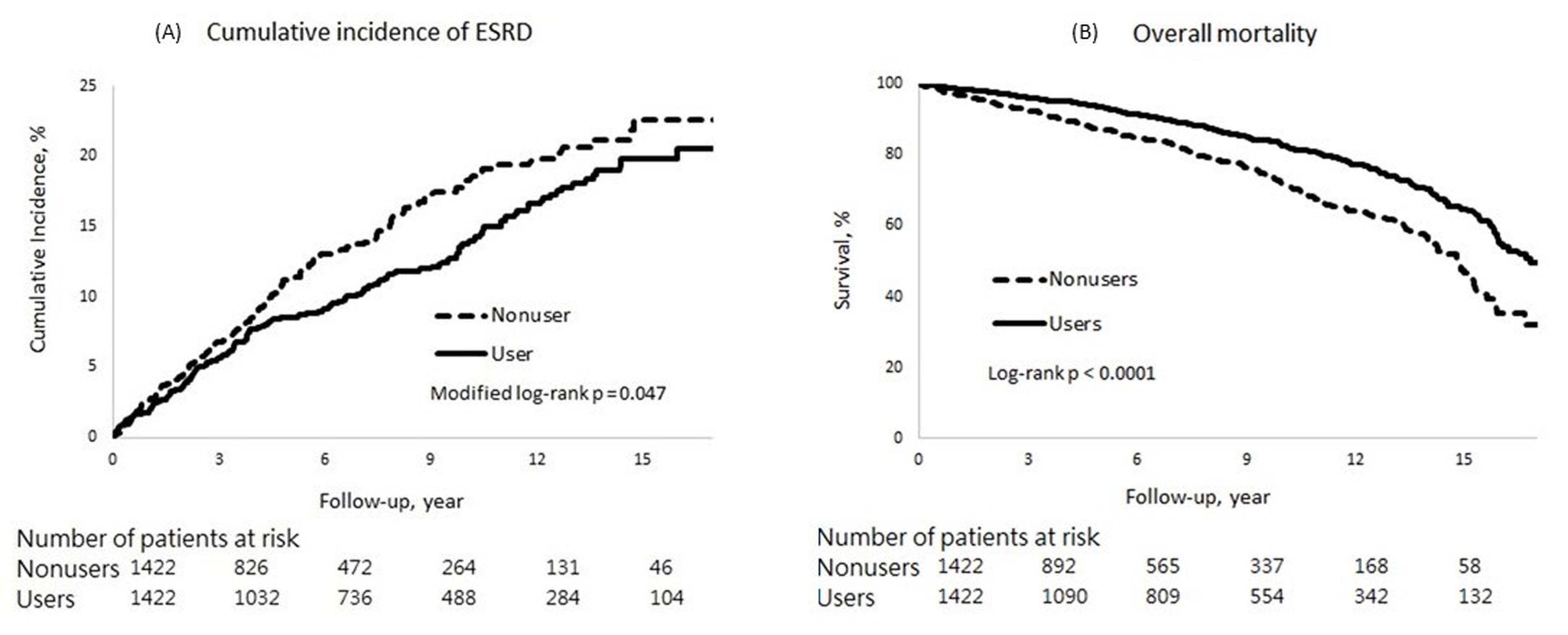

Supplement: Supplementary file 1 [file DataSheet1.doc]
